# Design of a Recombinant Multivalent Epitope Vaccine Based on SARS-CoV-2 and Its Variants in Immunoinformatics Approaches

**DOI:** 10.3389/fimmu.2022.884433

**Published:** 2022-05-06

**Authors:** Mingkai Yu, Yuejie Zhu, Yujiao Li, Zhiqiang Chen, Zhiwei Li, Jing Wang, Zheng Li, Fengbo Zhang, Jianbing Ding

**Affiliations:** ^1^ Department of Immunology, School of Basic Medical Sciences, Xinjiang Medical University, Urumqi, China; ^2^ Xinjiang Key Molecular Biology Laboratory of Endemic Disease, Xinjiang Medical University, Urumqi, China; ^3^ Reproductive Medicine Center, The First Affiliated Hospital of Xinjiang Medical University, Urumqi, China; ^4^ Department of Blood Transfusion, The First Affiliated Hospital of Xinjiang Medical University, Urumqi, China; ^5^ Clinical Laboratory Center, Xinjiang Uygur Autonomous Region People’s Hospital, Urumqi, China; ^6^ Xinjiang Laboratory of Respiratory Disease Research, Traditional Chinese Medicine Hospital Affiliated to Xinjiang Medical University, Urumqi, China; ^7^ Department of Clinical Laboratory, The First Affiliated Hospital of Xinjiang Medical University, Urumqi, China; ^8^ State Key Laboratory of Pathogenesis, Prevention, Treatment of Central Asian High Incidence Diseases, The First Affiliated Hospital of Xinjiang Medical University, Urumqi, China

**Keywords:** SARS-CoV-2, COVID-19, variant, vaccine, epitope, immunoinformatics

## Abstract

The development of an effective multivalent vaccine against SARS-CoV-2 variants is an important means to improve the global public health situation caused by COVID-19. In this study, we identified the antigen epitopes of the main global epidemic SARS-CoV-2 and mutated virus strains using immunoinformatics approach, and screened out 8 cytotoxic T lymphocyte epitopes (CTLEs), 17 helper T lymphocyte epitopes (HTLEs), 9 linear B-cell epitopes (LBEs) and 4 conformational B-cell epitopes (CBEs). The global population coverage of CTLEs and HTLEs was 93.16% and 99.9% respectively. These epitopes were spliced together by corresponding linkers and recombined into multivalent vaccine. In silico tests, the vaccine protein was a non-allergen and the docking with TLR-3 molecule showed a strong interaction. The results of immune simulation showed that the vaccine may be helpful to initiate both cellular and humoral immunity against all VOC. The optimistic immunogenicity of the vaccine was confirmed *in vivo* and *in vitro* finally. Therefore, our vaccine may have potential protection against SARS-CoV-2 and its variants.

## 1 Introduction

In December 2019, the world witnessed the first coronavirus pandemic in human history. The coronavirus is a novel virus which was named as the severe acute respiratory syndrome coronavirus 2 (SARS-CoV-2) by the International Committee on Taxonomy of Viruses (ICTV), and caused corona virus disease 2019 (COVID-19) which was named by the World Health Organization (WHO) (1). The SARS-CoV-2 can invade lung and other multiple organs, causing severe pathological damage (2). Most of the young adults have no serious clinical symptoms, but the elderly and patients with underlying diseases are more serious after infected by SARS-CoV-2 (3, 4). The human individual is generally vulnerable to the SARS-CoV-2. The Worldometer database showed that more than 240 million novel coronavirus pneumonia cases had been confirmed and the cumulative death toll was more than four million all over the world as of October 2021. The COVID-19 had triggered serious public health problem and additional financial burdens around the world (5, 6).

The SARS-CoV-2 is a single and positive stranded RNA virus with an enveloped structure (7). The length of the virus genome is 26-32 kb and diameter of virus particles is between 70-120 nm. The SARS-CoV-2 encodes replicase, spike protein (S protein), envelope protein (E protein), membrane protein (M protein) and nucleocapsid protein (N protein) successively in sequence from genome 5’ to 3’ end (8). The S protein, especially, plays a key role in SARS-CoV-2 infection for it can bind human angiotensin converting enzyme 2 (ACE2) to infect target cells. As RNA virus, SARS-CoV-2 mutation probability is much higher than DNA virus (9). Up to now, some mutants of SARS-CoV-2 have attracted great attention. They are uniformly called variants of human concern (VOC), because the transmission, virulence and sensitivity of VOC have changed significantly compared with the original strain (10).

The variant named B.1.1.7 first appeared in the United Kingdom and had 10 characteristic mutations in S protein, including D614G and N501Y mutations in ACE2 receptor binding domain (RBD) and two characteristic mutations in Protein N (11, 12). It was reported that the transmission rate of B.1.1.7 variant increased by 35-45% and the mortality of patients infected with B.1.1.7 variant can be increased by 30% (13, 14). The variant B.1.351 had three mutations combination of N501Y, E484K and K417N on RBD in South Africa and then spread around the world (15). It was later confirmed that the binding strength of B.1.351 variant to ACE2 was three times stronger than that of the wild type (16). The variant P.1 first reported in Brazil and Japan that derived from Lineage B.1.1.28, and it had 12 S protein missense mutations, including three RBD mutations of E484K, K417T and N501Y (17). Paiva et al. (18) recently found that P.1 variant existed in cases of reinfection with SARS-CoV-2, which suggested that P.1 can escape from the antibody produced by the infection of the original strain. The variant B.1.429 in California contained four missense mutations, of which the L452R mutation is located in RBD (11). Recently, researchers at the University of California found that the variant B.1.429 can affect the neutralization efficiency of protective antibodies, so the B.1.429 variant is more contagious in people. Los Alamos National Laboratory in New Mexico also found that B.1.1.7 and B.1.429 variant can recombine and fuse with each other, which warns us that people may have entered a new phase of the SARS-CoV-2 pandemic. The variant B.1.617.2 with 13 amino acid mutations was first discovered in India in October 2020, including E484Q, L452R and P681R in its spike protein (19). Therefore, the mutations weakened the binding between SARS-CoV-2 S protein and existing antibodies, and potentially reduced the protection of the vaccine (13, 20).

At present, there are many kinds of vaccines on the market globally, such as BioNTech vaccine (Pfizer Corp), mRNA vaccine (Moderna Corp), AZD1222 adenovirus-based vaccine (Astrazeneca Corp), JNJ-78436735 adenovirus-based vaccine (Johnson & Johnson Corp), NVX-CoV2373 nanoparticle-based vaccine (Novavax Corp), etc. However, with the emergence of SARS-CoV-2 variants, the epitopes of original virus have also changed, threatening the effectiveness of existing vaccines (21). In view of the variation characteristics of SARS-CoV-2, it is a particularly urge task to further develop efficient COVID-19 vaccine for preventing this serious infectious disease (22). Currently, it has been proved that the SARS-CoV-2 variants can escape the humoral immunity induced by the vaccine, so the development of specific vaccines against virus mutants is particularly important for the prevention and control of COVID-19 epidemic. The research and development of global vaccine involve a variety of technical schemes, among which the recombinant protein vaccine made *in vitro* is the most widely used (23). Recombinant protein vaccine can contain different specific pathogen proteins and stimulate human body to produce corresponding antibodies. Because the spike protein coupled with ACE2 receptor mediates the viral entry, it plays a crucial function in the SARS-CoV-2 infection and has been used the top candidate antigen for the development of vaccine. With the immunoinformatics, the activation of immunogenic epitopes on the S protein may be a more useful approach. We have researched scientifically rigorous strategy of multi-epitope peptides based on different proteins against parasitic and bacteria diseases, such as hydatid and brucellosis (24–28). In this study, we have used immunoinformatics to predict and design a multivalent and multi-epitope vaccine that derived from the S proteins of prevalent SARS-CoV-2 and its variants for conferring optimistic protection. 

## 2 Materials and Methods

### 2.1 The Acquisition of Vaccine Candidate Antigens

#### 2.1.1 Screening SARS-CoV-2 Variants

China National Center for Bio-information (CNCB) (https://bigd.big.ac.cn/ncov/variation/annotation) had classified the SARS-CoV-2 variants. This study was graded according to the assessment of CNCB website, and the Class I variants (the population incidence was greater than 0.05) were selected to screen antigens for vaccine design. Finally, we selected 5 SARS-CoV-2 variants of different lineages with the best quality evaluation.

#### 2.1.2 Obtaining S Protein Sequences

Because of the important role of S protein in the SARS-CoV-2 infecting host process, the Protein S of SARS-CoV-2 and its variants were used to identify antigen epitopes and to design vaccine. The S protein sequences of Wuhan-Hu-1 and B.1.1.7, B.1.351, P.1, B.1.429, B.1.617.2 etc. variants were obtained from National Center for Biotechnology Information (NCBI) (https://www.ncbi.nlm.nih.gov/). In addition, antigenicity of all S proteins was predicted using online software VaxiJen 2.0 (http://www.ddg-pharmfac.net/vaxijen/VaxiJen/VaxiJen.html). The VaxiJen was the first server that did not rely on protein sequence alignment and only classified proteins according to their physicochemical properties, so as to predict protective antigens (29).

#### 2.1.3 The Sequence Alignment of Protein S

The sequence alignment of all S proteins of Wuhan-Hu-1 and B.1.1.7, B.1.351, P.1, B.1.429 B.1.617.2 variants were performed using software SnapGene, which could screen out the mutated amino acid sites.

### 2.2 The Prediction of Signal Peptide

To determine whether there are signal peptide regions in the candidate antigen proteins, the signal peptides of S proteins in different SARS-CoV-2 lineages were predicted using SignalP-5.0 Server (http://www.cbs.dtu.dk/services/SignalP/) (30).

### 2.3 The Identification of Antigen Epitopes

#### 2.3.1 The Identification of CTL Epitopes

The antigenic epitopes were restrictive in binding to human leukocyte antigen (HLA) molecules. In order to make the vaccine effect cover the global population more widely, we expanded the selection of HLA allele. According to the HLA allele reference set from the IEDB website (http://tools.iedb.org/mhci/) database (31), there are 27 high frequency HLA-I alleles (HLA-A*01:01, HLA-A*02:01, HLA-A*02:03, HLA-A*02:06, HLA-A*03:01, HLA-A*11:01, HLA-A*23:01, HLA-A*24:02, HLA-A*26:01, HLA-A*30:01, HLA-A*30:02, HLA-A*31:01, HLA-A*32:01, HLA-A*33:01, HLA-A*68:01, HLA-A*68:02, HLA-B*07:02, HLA-B*08:01, HLA-B*15:01, HLA-B*35:01, HLA-B*40:01, HLA-B*44:02, HLA-B*44:03, HLA-B*51:01, HLA-B*53:01, HLA-B*57:01, HLA-B*58:01) were used to predict the SARS-CoV-2 cytotoxic T lymphocyte (CTL) antigenic epitopes. In this study, the online software NetCTLpan was used to predict the CTL epitopes (CTLEs). The online Server NetCTLpan 1.1 (http://www.cbs.dtu.dk/services/NetCTLpan/) used artificial neural networks (ANNs), and it can simulate the binding between antigen peptide and major histocompatibility complex (MHC) for the prediction of MHC class I antigenic epitopes (32). The signal peptide sequences were removed when predicting epitopes in S proteins. In each virus strain, the CTLEs that appeared in four HLA-I alleles at the same time were selected as the dominant epitopes. And the dominant epitopes of six virus strains were compared and integrated for vaccine design. The antigenicity of epitopes screened out was further predicted using VaxiJen 2.0, and the antigen epitopes that its antigenicity value greater than threshold were used for the construction of the final vaccine.

#### 2.3.2 The Identification of HTL Epitopes

According to the HLA allele reference set from IEDB (http://tools.iedb.org/mhcii/) (32), there were also 27 high frequency HLA-II alleles (HLA-DRB1*01:01, HLA-DRB1*03:01, HLA-DRB1*04:01, HLA-DRB1*04:05, HLA-DRB1*07:01, HLA-DRB1*08:02, HLA-DRB1*09:01, HLA-DRB1*11:01, HLA-DRB1*12:01, HLA-DRB1*13:02, HLA-DRB1*15:01, HLA-DRB3*01:01, HLA-DRB3*02:02, HLA-DRB4*01:01, HLA-DRB5*01:01, HLA-DQA1*05:01/DQB1*02:01, HLA-DQA1*05:01/DQB1*03:01, HLA-DQA1*03:01/DQB1*03:02, HLA-DQA1*04:01/DQB1*04:02, HLA-DQA1*01:01/DQB1*05:01, HLA-DQA1*01:02/DQB1*06:02, HLA-DPA1*02:01/DPB1*01:01, HLA-DPA1*01:03/DPB1*02:01, HLA-DPA1*01:03/DPB1*04:01,HLA-DPA1*03:01/DPB1*04:02, HLA-DPA1*02:01/DPB1*05:01, HLA-DPA1*02:01/DPB1*14:01) were used to predict the SARS-CoV-2 helper T lymphocyte (HTL) antigen epitopes. The online Server NetMHCIIpan 4.0 (https://services.healthtech.dtu.dk/service.php?NetMHCIIpan-4.0) (33) was used to predict the HTL epitopes (HTLEs). In each virus strain,the HTLEs that appeared in six HLA-II alleles at the same time were selected as the dominant epitopes and the prediction method of the final epitope was the same as that of CTLEs.

#### 2.3.3 The Identification of Linear B-Cell Epitopes

The online Server BepiPred-2.0 (https://services.healthtech.dtu.dk/service.php?BepiPred-2.0) (34) was used to predict the B-cell epitopes (BEs). The BEs were divided into linear epitopes and conformational epitopes. And the BepiPred-2.0 predicted that was linear B-cell epitopes (LBEs). The BEs of six virus strains were compared and synthesized, and the finally screened epitopes were used for vaccine construction. The signal peptide sequences were removed when predicting epitopes in S proteins.

#### 2.3.4 The Identification of Conformational B-Cell Epitopes

The online software IEDB (http://tools.iedb.org/ellipro/) was used to predict the conformational B-cell epitopes (CBEs). The predicted conformational epitopes of six virus strains were compared and synthesized. The predicted conformational epitopes of six virus strains were compared and synthesized, and finally used in the construction of vaccine. And the signal peptide sequences were removed when predicting epitopes in S proteins.

### 2.4 Design and Construction of Vaccine

All the filtered CTLEs, HLTEs, LBEs and CBEs were placed and used in the multivalent vaccine construct. In light of the research of Dong (35), our study selected reasonable Linker-sequence to connect the SARS-CoV-2 antigen epitopes respectively. First, the AAY linkers were inserted between CTLEs, the GPGPG linkers were inserted between HTLEs and the KK linkers were inserted between BEs. Then the TAT-sequence (TGALLAAGAAA) was attached to the C-terminal of merged epitopes, which can make the vaccine deliver intracellularly (36). The recombinant SARS-CoV-2 multivalent epitope vaccine was named as *rSMEV* in the study.

### 2.5 Secondary and Tertiary Structure Prediction of *rSMEV*

When the antigen epitopes were connected sequentially by Linkers, the secondary structure of *rSMEV* was predicted by online software SOMPA (npsa-prabi.ibcp.fr/cgi-bin/npsa_automat.pl?page=npsa_sopma.html). The SOMPA adopted the self-optimized prediction method (SOPM) to improve the accuracy of prediction by multiple alignment with protein sequences of the same family (37). And then the tertiary structure was predicted by software Rosettafold. The Rosettafold had ultra-high tertiary structure prediction accuracy by successively converting and integrating the three-track network information of 1D sequence, 2D distance and 3D coordinates (38). 

### 2.6 Structure Optimization and Quality Validation

After primary 3D modeling, the initial vaccine 3D model was further optimized by GalaxyRefine Server (http://galaxy.seoklab.org/). The GalaxyRefine adopted ab initio modeling, and refined the loop or terminus regions in the primary protein 3D model (39). At the same time, the rationality of the optimized protein tertiary structure needs to be further verified. The ProSA-web (https://prosa.services.came.sbg.ac.at/prosa.php) and the Structure Assessment service of SWISS-MODEL (https://swissmodel.expasy.org/assess) were used to verify the tertiary structure quality of vaccine construct. The ProSA-web could evaluate the overall model quality by Z-Score as well as the local model quality. In addition, the SWISS-MODEL had drawn Ramachandran plot that displayed favorable areas of backbone dihedral angles against amino acid residues in tertiary structure (40).

### 2.7 Analysis and Evaluation of *rSMEV*

#### 2.7.1 Analysis of *rSMEV* Physicochemical Properties

The online tool ProtParam (https://web.expasy.org/protparam/) (41) was used to predict the physical and chemical properties of *rSMEV*. The important physicochemical properties that ProtParam had performed included molecular weight, theoretical isoelectric point (PI), charged polar, atomic composition, half-life, stability, hydrophobicity and so on. According to the physicochemical properties of vaccine protein, it can guide the preparation of vaccine solution and the vaccination strategy *in vivo* experiment.

#### 2.7.2 Analysis of *rSMEV* Allergenicity and Antigenicity

In order to ensure the safety of the vaccine, we predicted its allergenicity. The online software AllerTOP 2.0 (http://www.ddg-pharmfac.net/AllerTOP/) was used to predict the allergenicity of *rSMEV*. This prediction tool is based on auto cross covariance (ACC) transformation of protein sequences into uniform equal-length vectors, and the target protein was compared with allergens and non-allergens data sets for predicting allergen properties (29). Meanwhile, the VaxiJen 2.0 (http://www.ddg-pharmfac.net/vaxijen/VaxiJen/VaxiJen.html) (34) was used to predict the antigenicity of *rSMEV*.

#### 2.7.3 Evaluation of *rSMEV* Population Coverage

The alleles and genotype frequencies were different among different populations in the world, and the polymorphism of HLA alleles affects the binding ability of antigen peptides to HLA-I or HLA-II molecules (42). The *rSMEV* had contained both HLA-I and HLA-II antigen epitopes. In addition, a wide range of HLA-I and HLA-II alleles were selected when predicting antigen epitopes. In order to verify the coverage of *rSMEV* to the world population, the IEDB (http://tools.iedb.org/population/) (32) was used to analyze the *rSMEV* population coverage.

### 2.8 Molecular Docking of *rSMEV* With TLR-3

The toll like receptor (TLR) was a class of important protein molecules involved in innate immunity, and it was also a bridge between nonspecific immunity and specific immunity (43). The TLR-3 molecule was found in a wide range of antigen presenting cells (APCs), such as tissue dendritic cells and monocytes, and TLR-3 could activate the specific recognition response of body cells to RNA virus infection (44). In the study, the binding affinity that *rSMEV* with TLR-3 was confirmed by the molecular docking approach. The tertiary structure of TLR-3 molecular was obtained from the PDB database (http://www.wwpdb.org/). The molecular docking works were performed by ClusPro Server (https://cluspro.bu.edu/home.php) (45). And the interaction interface residues analysis was performed by software PyMol and Ligplot.

### 2.9 Molecular Dynamics Simulation

After molecular docking, the best-docked system was solvated in a rectangular box of TIP3P (46) waters extending up to minimum cutoff of 15 Å from the protein boundary. Cl^-^ or Na^+^ ions were added into the protein surface to neutralize the total charges of the systems. The Amber ff14SB force field (47) was employed for the protein in all of the molecular dynamics (MD) simulations. The initial structures were fully minimized using combined steepest descent and conjugate gradient method. Then, the systems were gently annealed from 10 to 300 K under canonical ensemble for 0.2 ns with a weak restraint of 15 kcal/mol/Å. The 500 ps of density equilibration was performed under isothermal-isobaric ensemble at target temperature of 300K and the target pressure of 1.0 atm using Langevin-thermostat (48) and Berendsen barostat (49) with collision frequency of 0.002 ns and pressure-relaxation time of 0.001 ns. After proper minimizations and equilibrations, a productive MD run of 100 ns was performed for all the complex systems. The MD simulations were performed with GROMACS 2021.3 (50), and calculated MMGBSA through MMPBSA.py by Amber tools (51).

### 2.10 Immune Simulation

To detect the immune response of *rSMEV* to the host, the C-ImmSim Server (https://kraken.iac.rm.cnr.it/C-IMMSIM/) was used to perform the immune simulation (52). The C-ImmSim could simulate the cellular and humoral immunity in the immune response of vaccine to mammals. It provided a fast Position Specific Scoring Matrix (PSSM) to predict the epitopes that bound to HLA molecules (53). In this simulation work, the HLA heterozygous combination of host was HLA-A (HLA-A*01:01, HLA-A*03:01), HLA-B (HLA-B*15:01, HLA-B*35:01) and HLA-DR (HLA-DRB1*0401, HLA-DRB1*1302). The selection of host HLA was determined according to the highest frequency HLA molecules of all epitopes in *rSMEV*. The injection procedure was once every four weeks, a total of three injections. The time steps were 1, 84 and 168 (one time step corresponds to 8 h) (54). In addition, the Random Seed (12345), the Simulation Volume (10), the Adjuvant dose (100) and Number of antigens injected (1000) were system default. The simulation step was set as 1050 (53).

### 2.11 Codon Optimization

In order to better express the vaccine protein for subsequent animal *in vivo* experiment, we analyzed the adaptability and preference of the codon of *rSMEV*. The online tool JCat (JAVA Codon Adaptation Tool) (http://www.jcat.de/Start.jsp) was used to optimize the codon (55). The Escherichia coli (Strain K12) was chosen to express the *rSMEV* protein. Meanwhile, avoided rho-independent transcription terminators option, avoid prokaryotic ribosome binding sites option and avoided cleavage sites of restriction enzymes et al. options were selected for generating optimized DNA sequence corresponding to *rSMEV*.

### 2.12 In Silico Cloning of *rSMEV*

The pET-28a (+) was selected as the vector that expressed the *rSMEV* protein, and it was introduced into Escherichia coli. Before inserting the DNA sequence of *rSMEV* into the vector, it had been confirmed that there were no specific restriction enzyme recognition sites in the target gene sequence. Finally, the corresponding restriction endonuclease sequences were inserted at the N- and C-terminal of the DNA sequence of *rSMEV*. The in silico cloning work was performed by SnapGene. Finally, the *rSMEV* vaccine protein was synthesized and purified by SynPeptide Co Ltd (Shanghai, China).

### 2.13 *In Vivo* and *In Vitro* Experiments

#### 2.13.1 Animal

The eight-week-old SPF BALB/c mice provided by the Animal Experiment Center of Xinjiang Medical University were randomly divided into rSMEV and Healthy control (HC) two groups. The mice were dripped intranasally with vaccine protein in rSMEV group (n=8), and the mice was dripped intranasally with normal saline in HC group (n=8). The concentration of vaccine protein solution was 500 μg/mL, each mouse was given 60 μL each time (30 μL each nostril), once every 2 weeks, 3 times in total and the vaccine protein was detected as endotoxin-negative by limulus amebocyte lysate (LAL) before using. Two weeks after the third immunization, the mouse splenocytes were collected aseptically in rSMEV and HC groups for experimental detection. The study was approved by the animal ethics committee of Xinjiang Medical University, and the whole animal experiment process was carried out in strict accordance with the experimental requirements and operation guidelines of the animal ethics committee of Xinjiang Medical University.

#### 2.13.2 ELISPOT

The Enzyme-Linked Immunospot (ELISPOT) experiment was performed for evaluating the specific B-cell response in mice. First, the *rSMEV* vaccine protein solution (10 μg/mL) was coated on the ELISPOT plate and incubated overnight at 4 °C. Then the plate was washed for 5 times by Microplate washer (Thermo Fisher, FI-01620 Vantaa, Finland) and blocked with 200 μl RPMI-1640 medium (Sigma Aldrich, St. Louis, UA) containing 10% fetal calf serum (FCS) (Sigma) for 30 minutes at 37 °C. After the plate was washed by PBS containing 0.05% Tween 20, the vaccine protein and mice splenocytes suspension prepared (2×10^5^) were added into the ELISPOT plate3. Three repeat wells were set for each sample and the plate was incubated for 20 hours in a 37 °C humidified incubator with 5% CO2. After the plate was washed for 5 times, the biotinylated detecting antibody (anti-mouse IgG) was added and the plate was incubated for 2 hours at room temperature. After the plate was washed, the Streptavidin-ALP was added and incubated at room temperature for 1 hour. The BCIP/NBT solution (MlBio) was added and incubated for chromogenic reaction. The plate was rinsed and dried, the antibody secreting cell (ASC) spot were counted using EliSpot Reader (AID, D 72479, Germany). In this experiment work, the Mouse IgG ELISpot BASIC kit (ALP) (3825-2A, Mabtech, Stockholm, Sweden) was adopted for the ELISPOT experiment.

#### 2.13.3 ELISA

The enzyme-linked immunosorbent assay (ELISA) experiment was performed for evaluating the CD4^+^ T-cell response in mice. First, the splenocytes in HC and rSMEV groups were co-cultured with the rSMEV vaccine protein (5 μg/mL). The splenocytes were incubated with RPMI-1640 medium containing 10% FCS for 48 hours in a 37 °C under 5% CO_2_ humidified incubator. Then the culture supernatant was collected, and the samples and standards were added into the IFN-γ and IL-4 ELISA plates after plates were washed. The ELISA plates were incubated at 37 °C for 1.5 hours and washed for 5 times. The biotinylated detecting antibody was added to the plates and incubated at 37 °C for 1 hour. After the plates were washed for 5 times, the Streptavidin-ALP was added and incubated at 37 °C for 30 minutes. The BCIP/NBT solution were added for chromogenic reaction at 37 °C away from light for 15 minutes. Finally, the termination solution was added, and the OD450nm value was measured immediately by Spectrophotometer (Thermo Fisher, FI-01620 Vantaa, Finland) for detecting the corresponding level of IFN-γ and IL-4. The Mouse IFN-γ ELISA Kit (SEKM-0031, Solarbio Science & Technology Co., Ltd, Beijing, China) and Mouse IL-4 ELISA Kit (SEKM-0005, Solarbio Science & Technology Co., Ltd, Beijing, China) were adopted for ELISA experiment. 

## 3 Results

**Figure 1:**
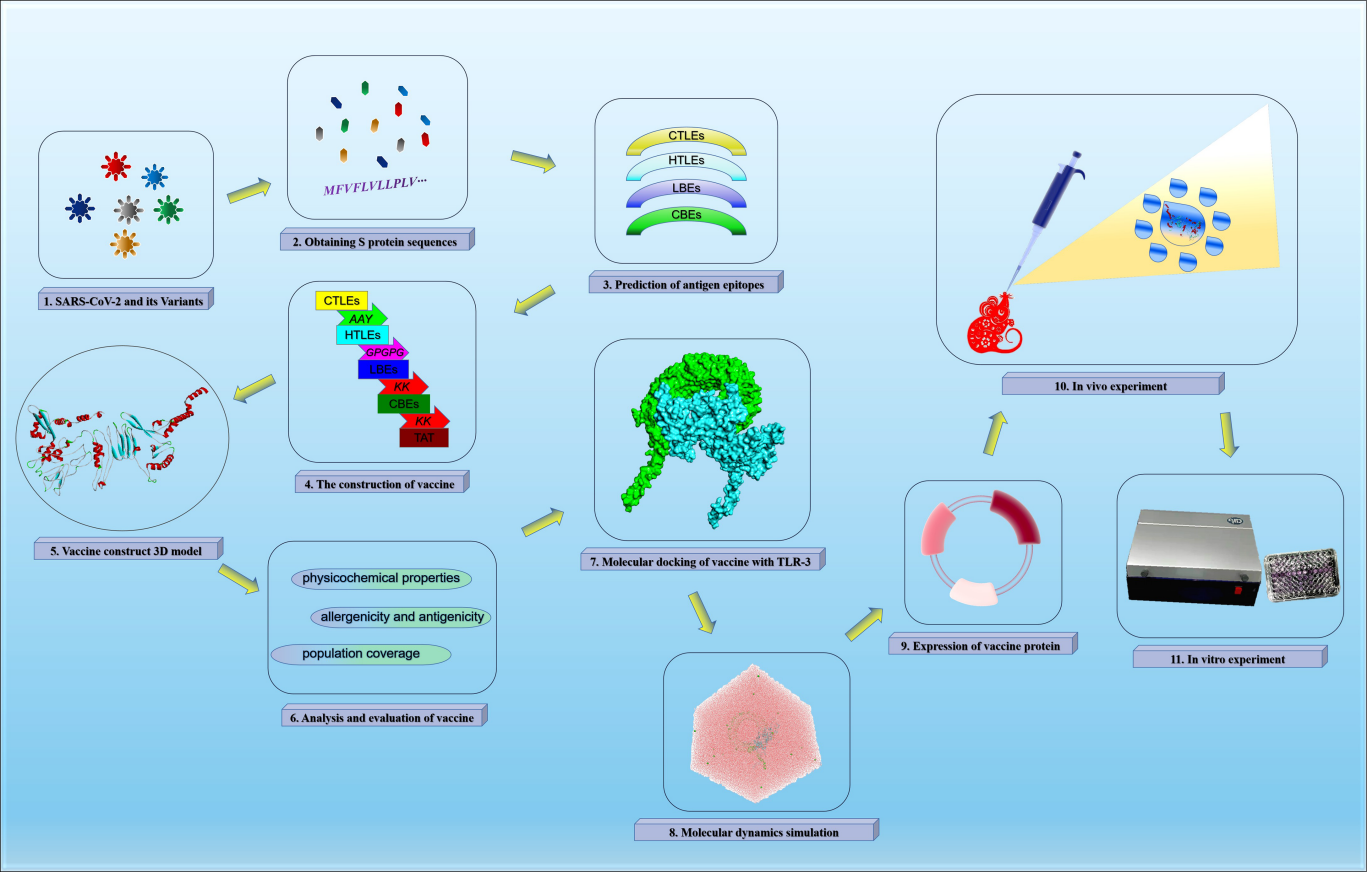
The vaccine construction strategy.

The vaccine construction strategy of this study was shown in **Figure 1**.

### 3.1 The Acquisition of Vaccine Candidate Antigens

#### 3.1.1 Screening SARS-CoV-2 Variants

The GenBank search number of the six SARS-CoV-2 virus strains was NC_045512 (Wuhan-Hu-1), MW642026 (Lineage B.1.1.7), MW621453 (Lineage B.1.351), MW642250 (Lineage P.1), MW631888 (Lineage B.1.429) and MZ491545 (Lineage B.1.617.2) separately.

#### 3.1.2 Obtaining S Protein Sequences

The GenBank accession number of S protein derived from six SARS-CoV-2 virus strains was YP_009724390 (Wuhan-Hu-1), QRX39358 (Lineage B.1.1.7), QRV12312 (Lineage B.1.351), QRX39425 (Lineage P.1), QRW35522 (Lineage B.1.429) and QXF88313 (Lineage B.1.617.2). The result was shown in **Table 1** and the amino acid sequence of these proteins was shown in **Supplementary Data**. 

**Table 1:** The antigenic value of SARS-CoV-2 S proteins.

Lineage	Protein	Accession number ^a^	Residue length	Predictive value ^b^
Wuhan-Hu-1	S	YP_009724390	1273aa	0.4646
B.1.1.7	S	QRX39358	1270aa	0.4742
B.1.351	S	QRV12312	1270aa	0.4656
P.1	S	QRX39425	1273aa	0.4723
B.1.429	S	QRW35522	1273aa	0.4783
B.1.617.2	S	QXF88313	1271aa	0.4695

a The accession number from National Center for Biotechnology Information (NCBI).

b The Virus was selected as the protective antigen prediction model in VaxiJen 2.0, and the default threshold of protective antigen prediction is 0.4.

#### 3.1.3 The Sequence Alignment of Protein S

**Figure 2:**
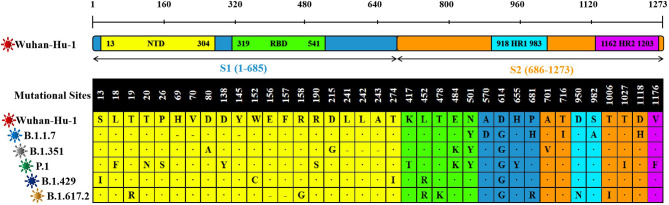
The sequence alignment of S proteins in different virus strains. The study had screened S proteins of six kinds of different SARS-CoV-2 virus strains. The Wuhan-Hu-1 strain was selected as the reference sequence in the sequence alignment. In this figure, we had used different colors to represent the different domains of the S protein. From the N-terminal to the C-terminal of the S protein, a total of 37 amino acid positions had been changed. Among these variations, “·” was omission, which represented the same amino acid as the reference sequence. “-” was loss, which represented the deletion of amino acid sequence. And the remaining mutation sites were amino acid missense mutation.

Compared with the original SARS-CoV-2 virus strain, the five virus variants had a total of 37 mutation sites. There were 25 mutation sites in the SARS-CoV-2 S1 region, among them, 20 mutation sites were in N-terminal domain (NTD) and five mutation sites in RBD. There were 17 mutation sites in the SARS-CoV-2 S2 region. Among them, two mutation sites were in heptad repeat region 1 (HR1) and one mutation site in HR2 (**Figure 2**).

### 3.2 The Prediction of Signal Peptide

**Figure 3:**
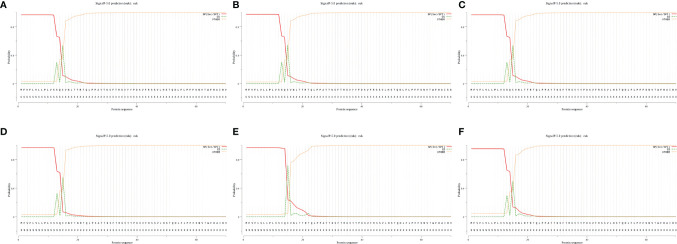
The signal peptides of S proteins. **(A)** The probability of signal peptide is 96.886%. **(B)** The probability of signal peptide is 97.653%. **(C)** The probability of signal peptide is 96.886%. **(D)** The probability of signal peptide is 96.685%. **(E)** The probability of signal peptide is 96.625%. **(F)** The probability of signal peptide is 95.053%. Because the virus strains Wuhan-Hu-1 and B.1.351 have high homology, and the server can only give the signal peptide probability of the first 60 amino acid residues, so **(A)** and **(C)** are very similar.

After the analysis, the S proteins of six different SARS-CoV-2 lineages all had signal peptide regions. The signal peptide region of Lineage Wuhan-Hu-1 was 1-15 (MFVFLVLLPLVSSQC), the signal peptide region of Lineage B.1.1.7 was 1-15 (mfvflvllplvssqc), the signal peptide region of Lineage B.1.351 (mfvflvllplvssqc), the signal peptide region of Lineage P.1 was (mfvflvllplvssqc), the signal peptide region of Lineage B.1.429 was 1-15 (mfvflvllplvsiqc), the signal peptide region of Lineage B.1.617.2 was (mfvflvllplvssqc) (**Figure 3**).

### 3.3 The Identification of Vaccine Antigen Epitopes

#### 3.3.1 The Identification of CTL Epitopes

**Figure 4:**
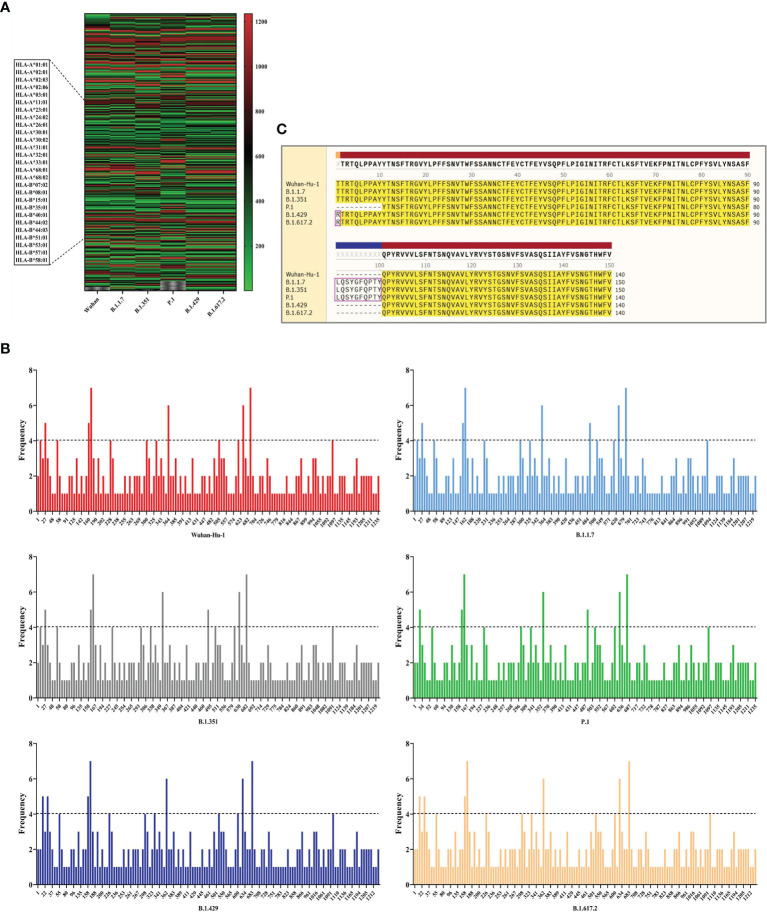
The identification of CTL epitopes. **(A)** After identifying the 27 kinds of HLA-I alleles, all the CTLEs of the S protein in the 6 SARS-CoV-2 strains were shown here. The abscissa represents the six virus strains and the ordinate represents the position of the beginning of the epitope peptide. **(B)** The abscissa represented the position of the beginning of the epitope, and the ordinate represented the frequency of the epitope in the 27 alleles. **(C)** All epitopes of each virus strain were connected in sequence according to the size of the starting position, and the epitopes that had undergone mutation were in the “pink” box. Through sequence alignment, all the common and variant epitopes of all strains were used for vaccine construction.

In this part, the CTLEs of six SARS-CoV-2 virus strains were analyzed by online Server NetCTLpan 1.1 with each virus strain having 27 kinds of CTLEs of HLA-I alleles. The Rank of CTLEs was less than 1% in the prediction using IEDB. All CTLEs were ranked from small to large according to the sequence start position (**Figure 4A**). For each virus strain, the CTLEs that appeared in four HLA-I alleles at the same time were selected as the dominant CTLEs (**Figure 4B**). And the dominant CTLEs of six virus strains were compared and integrated for vaccine design (**Figure 4C**). The antigenicity of epitopes screened out was further predicted, and the antigen epitopes that antigenicity value greater than the threshold were used for the construction of the final vaccine (**Supplementary Table 1**).

#### 3.3.2 The Identification of HTL Epitopes

**Figure 5:**
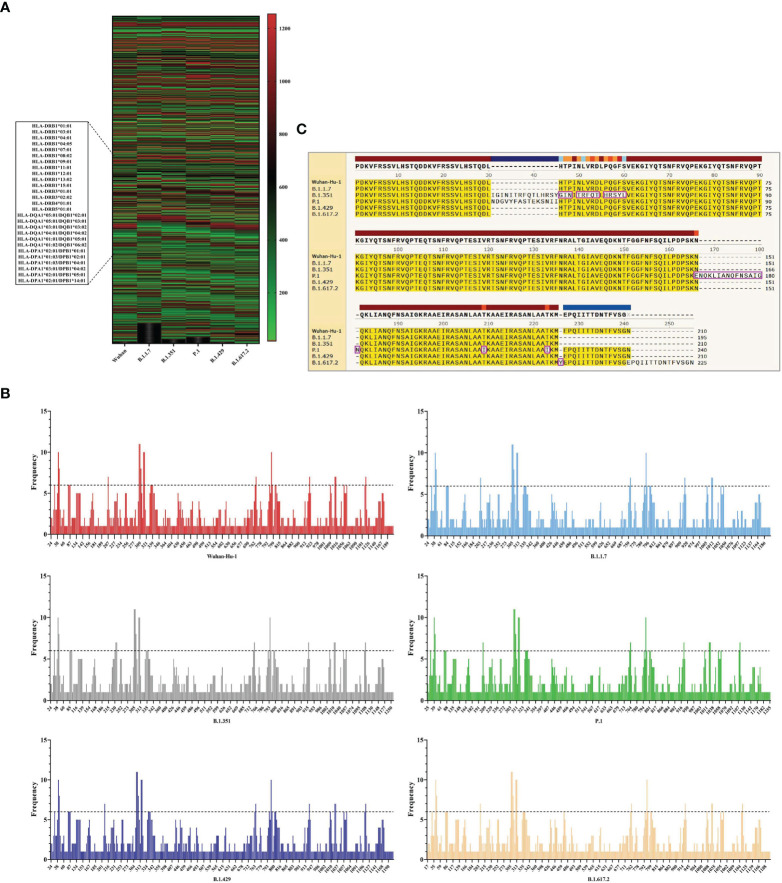
The identification of HTL epitopes. **(A)** After identifying the 27 kinds of HLA-II alleles, all the HTLEs of the S protein in the 6 SARS-CoV-2 strains were shown here. The abscissa represents the six virus strains and the ordinate represents the position of the beginning of the epitope peptide. **(B)** The abscissa represented the position of the beginning of the epitope, and the ordinate represented the frequency of the epitope in the 27 alleles. **(C)** All epitopes of each virus strain were connected in sequence according to the size of the starting position, and the epitopes that had undergone mutation were in the “pink” box. Through sequence alignment, all the common and variant epitopes of all strains were used for vaccine construction.

The HTLEs of six SARS-CoV-2 virus strains were analyzed by online Server NetMHCIIpan 4.0 and each virus strain also had 27 kinds of HTLEs of HLA-II alleles. The Rank of HTLEs was less than 2% in the prediction using IEDB. All HTLEs were ranked from small to large according to the sequence start position (**Figure 5A**). For each virus strain, the HTLEs that appeared in six HLA-II alleles at the same time were selected as the dominant HTLEs (**Figure 5B**). And the dominant HTLEs of six virus strains were compared and integrated for vaccine design (**Figure 5C**). The antigenicity of epitopes screened out was further predicted, and the antigen epitopes that antigenicity value greater than the threshold were used for the construction of the final vaccine (**Supplementary Table 2**).

#### 3.3.3 The Identification of Linear B-Cell Epitopes

**Figure 6:**
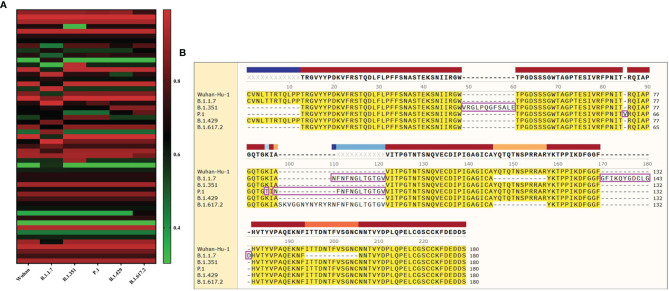
The identification of B-cell epitopes. **(A)** All the LBEs of the S protein in the 6 SARS-CoV-2 strains were shown here. The abscissa represents the six virus strains and the ordinate represents the Rank of the epitope peptide. **(B)** All epitopes of each virus strain were connected in sequence according to the size of the starting position, and the epitopes that had undergone mutation were in the “pink” box. Through sequence alignment, all the common and variant epitopes of all strains were used for vaccine construction.

The BepiPred-2.0 predicted the LBEs. In the result outputted from BepiPred-2.0, when the threshold of the peptide was greater than 0.8, the peptide sequence was recognized as an epitope (**Figure 6A**). The BEs of six virus strains were compared and synthesized, and the finally screened epitopes were identified as the dominant LBEs (**Figure 6B**). After all dominant LBEs were predicted for their antigenicity, the antigen epitopes that antigenicity value greater than the threshold were used for the construction of the final vaccine (**Supplementary Table 3**).

#### 3.3.4 The Identification of Conformational B-Cell Epitopes

**Figure 7:**
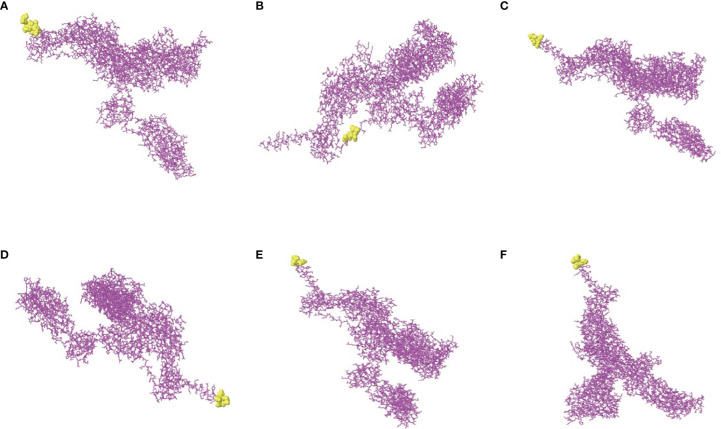
The conformational B-cell epitopes of six SARS-CoV-2 lineages. **(A)** The “yellow” region is the predicted best conformational B-cell epitope of S protein of virus strain Wuhan-Hu-1. **(B)** The “yellow” region is the predicted best conformational B-cell epitope of S protein of virus strain B.1.1.7. **(C)** The “yellow” region is the predicted best conformational B-cell epitope of S protein of virus strain B.1.351. **(D)** The “yellow” region is the predicted best conformational B-cell epitope of S protein of virus strain P.1. **(E)** The “yellow” region is the predicted best conformational B-cell epitope of S protein of virus strain B.1.429. **(F)** The “yellow” region is the predicted best conformational B-cell epitope of S protein of virus strain B.1.617.2.

The software IEDB had predicted the CBEs of S proteins from different SARS-CoV-2 lineages. We selected the best ranked CBEs among the six virus strains (**Figure 7**). After comparison, the sequences of CBEs were used to the construction of final vaccine (**Supplementary Table 4**).

### 3.4 Design and Construction of Vaccine

**Figure 8:**
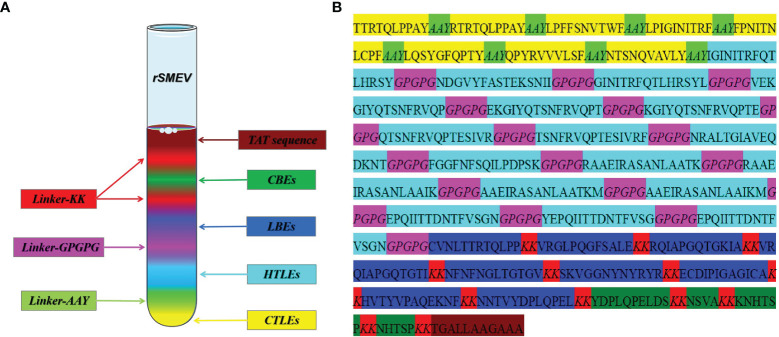
The design and construction of *rSMEV*. **(A)** Schematic diagram of all the components needed in vaccine construction, and the different colors represented different epitopes and linkers. **(B)** The specific amino acid sequence of all the epitopes and linkers required for vaccine construction, and the color of each part corresponded to the color in the **(A)**.

The *rSMEV* construct contained the best candidate antigen epitopes. There were 8 CTLEs, 17 HTLEs, 9 LBEs and 4 CBEs in the *rSMEV*. The Linker-AAY had connected all CTLEs, which can enhance the presentation of epitopes and help to produce suitable sites for antigen epitopes to bind to TAP transporters (35). The Linker-GPGPG had connected all HTLEs, the Linker-KK had connected all LBEs and CBEs (**Figure 8**). All the antigen epitopes for construction of *rSMEV* were shown in the **Table 2**. The number of vaccine residues of final designed multivalent vaccine was 614 aa.

**Table 2:** The antigen epitopes for vaccine construction.

NO.	Antigen epitope	Variant epitope	Type	Variation rate ^a^
1 2 3 4 5 6 7 8	TTRTQLPPAY RTRTQLPPAY LPFFSNVTWF LPIGINITRF FPNITNLCPF LQSYGFQPTY QPYRVVVLSF NTSNQVAVLY	RTRTQLPPAY LQSYGFQPTY	CTLEs	25.0%
9 10 11 12 13 14 15 16 17 18 19 20 21 22 23 24	IGINITRFQTLHRSY NDGVYFASTEKSNII GINITRFQTLHRSYL VEKGIYQTSNFRVQP EKGIYQTSNFRVQPT KGIYQTSNFRVQPTE QTSNFRVQPTESIVR TSNFRVQPTESIVRF NRALTGIAVEQDKNT FGGFNFSQILPDPSK RAAEIRASANLAATK RAAEIRASANLAAIK AAEIRASANLAATKM AAEIRASANLAAIKM EPQIITTDNTFVSGN YEPQIITTDNTFVSG	IGINITRFQTLHRSY NDGVYFASTEKSNII GINITRFQTLHRSYL RAAEIRASANLAAIK AAEIRASANLAAIKM YEPQIITTDNTFVSG	HTLEs	37.5%
25 26 27 28 29 30 31 32 33	CVNLTTRTQLPP VRGLPQGFSALE RQIAPGQTGKIA VRQIAPGQTGTI NFNFNGLTGTGV SKVGGNYNYRYR ECDIPIGAGICA HVTYVPAQEKNF NNTVYDPLQPEL	VRGLPQGFSALE VRQIAPGQTGTI NFNFNGLTGTGV	LBEs	33.3%7
34 35 36 37	YDPLQPELDS NSVA KNHTSP NHTSP	NSVA KNHTSP NHTSP	CBEs	75.0%

a Among the corresponding types of epitopes, the proportion of epitopes appearing in the virus variant.

### 3.5 Secondary and Tertiary Structure Prediction of *rSMEV*

**Figure 9:**
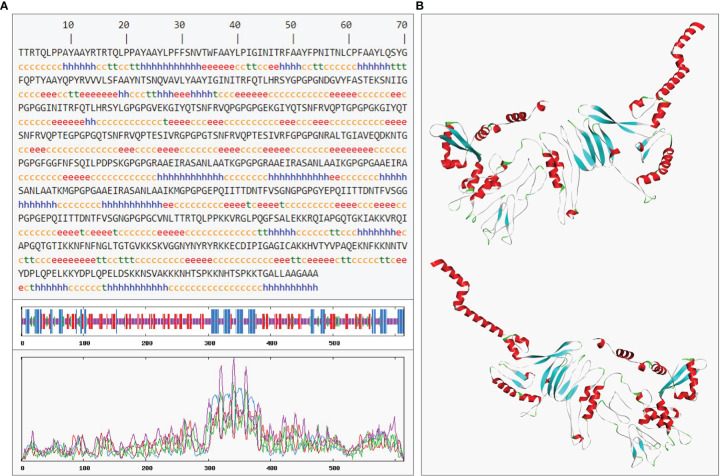
The prediction of *rSMEV* secondary and tertiary structure. **(A)** In the prediction of *rSMEV* secondary structure, the “h” represented the Alpha helix, the “e” represented the Extended strand, the “t” represented the Beta turn and the “c” represented the Random coil. **(B)** In this 3D model, the tertiary structure was displayed in front view and back view. The “red” part was Alpha helix, the “cyan” part was Extended strand, the “green” pare was Beta turn and the “gray” part was Random coil in 3D model.

In the result of secondary prediction, there were 20.03% Alpha helix (Hh), 23.94% Extended strand (Ee), 6.51% Beta turn (Tt) and 49.51% Random coil (Cc) (**Figure 9A**). The tertiary structure was shown in secondary structure style (**Figure 9B**), and the proportion of Hh, Ee, Tt and Cc was consistent with the secondary structure. This indirectly showed that the prediction of secondary structure and tertiary structure was reasonable.

### 3.6 Tertiary Structure Optimization and Quality Validation

**Figure 10:**
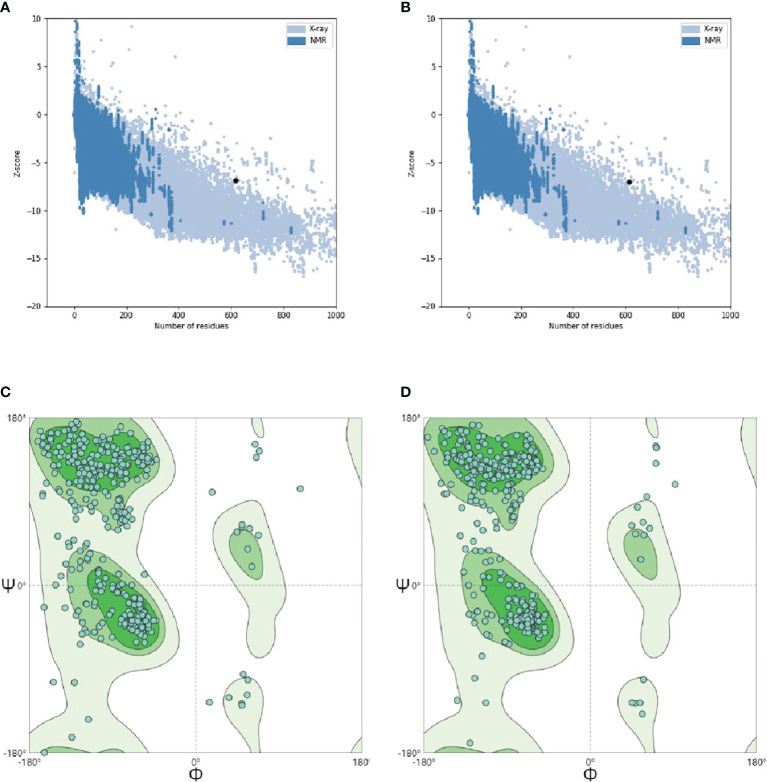
Tertiary structure optimization and quality validation. **(A)** and **(B)** are the Z-score evaluation results. The abscissa represents the number of amino acids of the protein and the ordinate represents the score. The region formed by blue and gray blots in the figure are the reasonable scoring areas of the protein, and the black dot represents the target protein. The good target protein with the corresponding number of amino acids should fall in reasonable area. **(C)** and **(D)** are Ramachandran plot. In the figure, dark green is favored regions, green is additional allowed regions, light green is generously allowed regions and white is disallowed regions. The rationality of amino acid residues in these regions decreased in turn. When evaluating the quality of 3D model, it is hoped that the proportion of amino acid residues in favored regions is larger.

After the initial tertiary structure was refined by GalaxyRefine Server, the server had outputted five optimized models. All models were further verified by ProSA-web, the website had calculated the Z-Score of models. The Z-Score of initial *Model* was −6.89, the optimized *Model 1* was −6.88, the optimized *Model 2* was −6.96, the optimized *Model 3* was −6.93, the optimized *Model 4* was −7.02 and the optimized *Model 5* was also −6.87. Through observation, it was found that the optimized *Model* 4 was more in line with the Z-score range of similar protein size. So, the optimized *Model* 4 was selected as the final tertiary structure. The Ramachandran plot drawn by SWISS-MODEL showed that there were 91.03% Ramachandran favored region, 2.45% Ramachandran Outliers region and 0.00% Rotamer Outliers region in initial structure, and there were 92.65% Ramachandran favored region, 1.47% Ramachandran Outliers region and 0.21% Rotamer Outliers region in final structure (**Figure 10**).

### 3.7 Analysis and Evaluation of *rSMEV*

#### 3.7.1 Analysis of *rSMEV* Physicochemical Properties

The vaccine protein had 614 amino acids, the total number of atoms was 9122 and the formula was C_2904_H_4540_N_822_O_850_S_6_. The molecular weight was 65 KD, and it was an acceptable vaccine since that the molecular weight of protein less than 110 KD could be easily purified (56). The theoretical pI was 10.04, and it included 29 acidic (negatively charged residues) amino acids (Asp + Glu) and 69 alkaline (positively charged residues) amino acids (Arg + Lys). The instability index (II) was computed to be 27.49 (when it<40, the protein is stable) (57), so the vaccine was classified as stable protein. The aliphatic index was 64.28 and Grand average of hydropathicity (GRAVY) was −0.481 (the range of GRAVY is −2∼2, negative value means that protein is hydrophilic) (58), so the vaccine belonged to hydrophilic protein. 

#### 3.7.2 Analysis of *rSMEV* Allergenicity and Antigenicity

The online software AllerTOP 2.0 defined the *rSMEV* as non-allergen, the UniProtKB accession number of the protein nearest to *rSMEV* was P46379 and P46379 was also probable non-allergen. The VaxiJen 2.0 showed that the overall prediction value for the protective antigen was 0.6858, the Virus was selected as the prediction model and the threshold for this model was 0.4. Therefore, in this allergenicity and antigenicity analysis, the *rSMEV* was identified as safe protective antigen.

#### 3.7.3 Evaluation of *rSMEV* Population Coverage

In the analysis of population coverage, the Class I and Class II T-cell epitopes were calculated separately. The IEDB showed that the HLA-I T-cell epitope covered 93.16% and HLA-II T-cell epitope covered 99.9% world population in the *rSMEV*. The detailed results were showed in **Supplementary Tables 5, 6** and **Supplementary Figure 1**.

### 3.8 Molecular Docking of *rSMEV* With TLR-3

**Figure 11:**
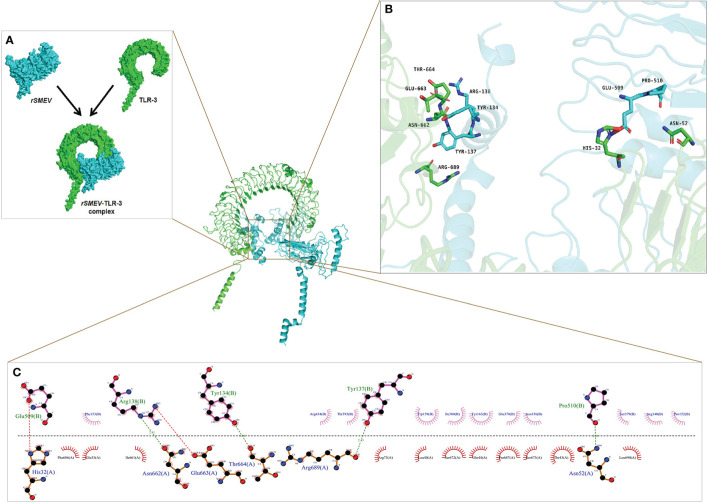
The molecular docking of *rSMEV* with TLR-3. **(A)** Schematic diagram of docking between vaccine molecule *rSMEV* and antigen recognition molecule TLR-3. The *rSMEV* is light blue and TLR-3 is green. After docking, the *rSMEV*-TLR-3 complex was formed. **(B)** The interaction interface predicted by the PyMol is three-dimensional. In this three-dimensional interaction interface, it is mainly ionic bond and hydrogen bond that participate in the interaction. **(C)** The interaction interface predicted by the Ligplot is two-dimensional. And in this two-dimensional interaction interface, the red dotted line represents ionic bond and the green dotted line represents hydrogen bond, so there are 2 ionic bonds and 4 hydrogen bonds involved in the interaction.

The PDB ID of TLR-3 was 7c76 (59) in the PDB database and the TLR-3 molecular obtained was a complex that blended with UNC93B1. The TLR-3 was isolated from the complex using software Discovery Studio for molecular docking. In this molecular docking work, the TLR-3 molecules acted as the receptor, while the *rSMEV* acted as ligand. The ClusPro Server can output some clusters after docking work, and each cluster has a different number of members. In the *rSMEV*-TLR-3 complex formed by the combination of vaccine molecule *rSMEV* and antigen recognition molecule TLR-3, the lowest complex energy weighted score was −1175.3 cal/mol and all the docking results were shown in **Supplementary Table 7**. The interaction interface residues analysis from PyMol was 3D and Ligplot was 2D format. The results show that there were two ionic bonds and four hydrogen bonds at the docking interface, which participated in the interaction between the subunits of the complex (**Figure 11**).

### 3.9 Molecular Dynamics Simulation

**Figure 12:**
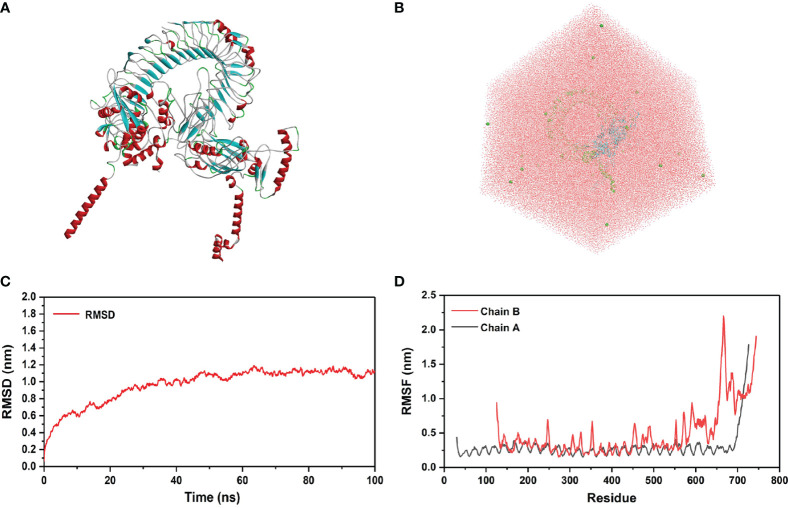
Molecular docking of *rSMEV* with TLR-3. **(A)** This is a three-dimensional model of *rSMEV* and TLR-3 complex, and its color is displayed in the form of secondary structure style. **(B)** This is the rectangular box of TIP3P waters, and it extends up to minimum cutoff of 15 Å from the protein boundary. After molecular docking, the best-docked system was solvated in it. **(C)** This is the RMSD running diagram of MD simulation. The abscissa represents the running time of MD simulation, and the ordinate is the value of RMSD. **(D)** This is the RMSF running diagram of MD simulation. The chain A is TLR-3 and the chain B is *rSMEV*. The abscissa represents each amino acid residue in the complex, and the ordinate represents the RMSF value.

In order to evaluate the structural stability of *rSMEV*-TLR-3 complex, the best docking pose (**Figure 12A**) of *rSMEV* and TLR-3 was simulated by MD. The docking complex was solvated in a rectangular box of TIP3P waters (**Figure 12B**). After running the MD simulation of 100 ns, the results showed that the root mean square displacement (RMSD) value rose sharply to 1nm within 30 ns. After 30 ns, the RMSD value tended to be stable, and the floating range of the overall operation was about 1 nm (**Figure 12C**). The root mean square mobility (RMSF) value showed that the two chains of the complex had a lower value (<1 nm) between residues 0-650, while the residues after site 650 had a larger RMSF value (>1 nm) (**Figure 12D**). Next, the average binding free energy was −88.54 ± 6.91 kcal/mol of complex detected by MMPBSA tool of the GROMACS. (**Supplementary Table 8**).

### 3.10 Immune Simulation

**Figure 13:**
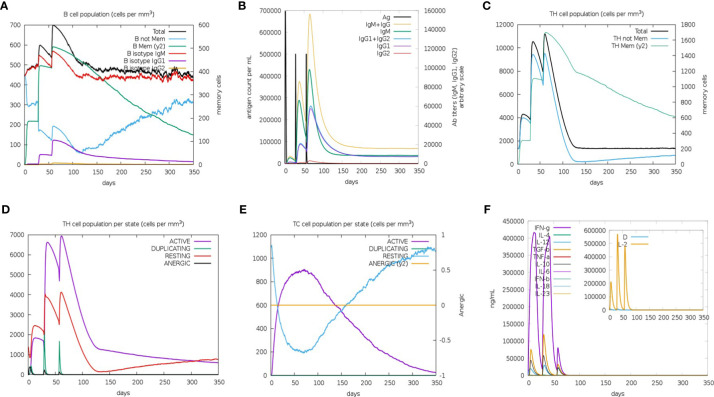
The immune simulated response spectrum. **(A)** The B-cell populations that could produce antibodies of various subtypes after vaccination. **(B)** The production of antibodies that represented the proliferation of the immune response after vaccination, and the various subtypes of immunoglobulins were shown in different colors. **(C)** The generation of memory and non-memory HTL populations after vaccination. **(D)** The HTL populations in various states. **(E)** The CTL populations in various states. **(F)** The cytokine profile showed the production of various cytokines after vaccination.

The C-ImmSim Server conducted the immune stimulation of the *rSMEV* vaccine. The Immune simulation results showed that the antigen count decreased with the increase of antibody level in the immune response, which was mainly due to the production of total B-lymphocytes (**Figure 13A**) and T-lymphocytes. The IgM + IgG and IgM antibodies were found in the primary immunization. And the levels of IgM + IgG, IgM, IgG1 and IgG1 + IgG2 antibodies increased in the secondary and tertiary immune responses compared to primary immune response (**Figure 13B**). Besides B-cell, total and memory TH-cell (helper T cell) (**Figure 13C**) populations along with active TH-cell (**Figure 13D**) populations increased during the secondary and tertiary immune responses. The active TC-cell (cytotoxic T lymphocyte) count was sustained growth after each immunization (**Figure 13E**). And in the three stages after immunization, the level of IFN-γ had increased (**Figure 13F**).

### 3.11 Codon Optimization

The *rSMEV* protein had 614 amino acids, and the DNA sequence of *rSMEV* had 1842 nucleotides after adaptability and preference analysis of codon. The codon adaptation index (CAI) of the improved sequence was 0.93. When the CAI-Value>0.8, the codon improved was considerate to have a high adaptability to the DNA sequence (**Supplementary Figure 2**). The GC-Content of the improved codon was 54.51% and the GC-Content of *Escherichia coli* strain K12 used in the study was 50.73%.

### 3.12 In Silico Cloning of *rSMEV*

The DNA sequence of *rSMEV* was inserted to the sites between XhoI (site 158) and BamHI (site 2006) in the pET-28a (+) vector. The DNA sequence of *rSMEV* was written in **Supplementary Data**. The XhoI and BamHI were common restriction endonucleases in molecular cloning, and the DNA sequence did not contain these two digestion sites (**Supplementary Figure 3**). 

### 3.13 The Results of ELISPOT and ELISA Experiments

**Figure 14:**
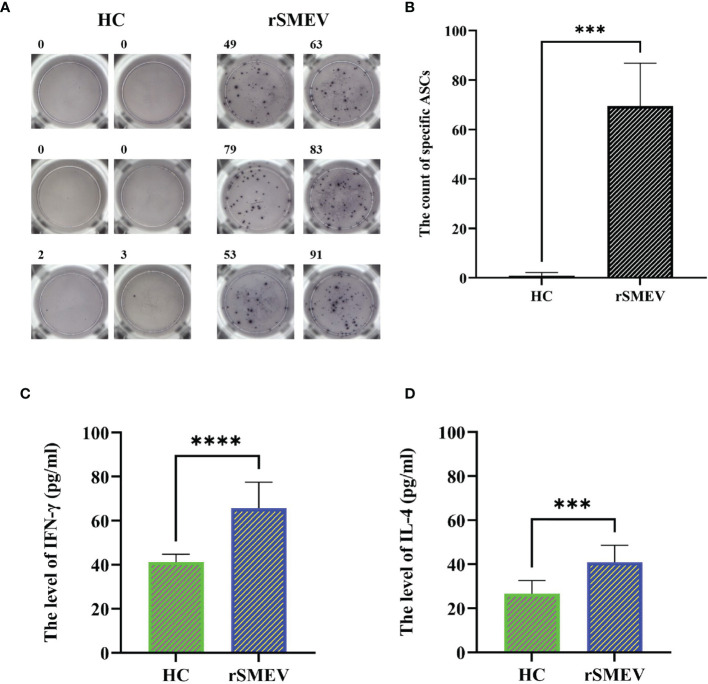
The results of ELISPOT and ELISA experiments. **(A)** The representative ELISPOT spot diagram in HC and rSMEV groups. **(B)** The count of ASCs in the HC and rSMEV groups were significantly different (***P<0.001) after using the two independent sample *t*-test, and the statistical test was two-tailed. The t-value was 9.671 and the degree of freedom was 5.059 (***P < 0.001). **(C)** The two independent sample *t*-test of the level of IFN-γ between HC group and rSMEV group, and the difference was statistically significant (****P<0.0001), the t-value was 6.247 and the degree of freedom was 18. **(D)** The two independent sample *t*-test of the level of IL-4 between HC group and rSMEV group, and the difference was statistically significant (***P<0.001), the t-value was 4.652 and the degree of freedom was 18.

In the study, the ELISPOT experiment was performed to evaluate the *rSMEV* induced B-cell response. The splenocytes of rSMEV group mice could secrete specific antibodies induced by vaccine protein, while the splenocytes of HC group mice did not secrete specific antibodies (**Figure 14A**). After statistical test, the specific antibody secreting cells (ASCs) in rSMEV group was significantly higher than that in HC group, and the difference was statistically significant (*P*<0.001) (**Figure 14B**). This suggested that *rSMEV* protein could activate B-cell reaction. The ELISA experiment was performed to evaluate the *rSMEV* induced CD4^+^ T-cell response. The CD4^+^ T-cell was activated by vaccine protein, so the content of IFN-γ (**Figure 14C**) and IL-4 (**Figure 14D**) in rSMEV group was significantly higher than that in HC group, and the difference was statistically significant (*P-*value of IFN-γ was less than 0.0001 and *P-*value of IL-4 was less than 0.001). This suggested that *rSMEV* protein could activate CD4^+^ T-cell reaction. 

## 4 Discussion

As you known, the decrease of vaccine protection ability elicited mainly by S protein mutation of SARS-CoV-2. There was no a multivalent epitope vaccine for S protein to resist the infection of SARS-CoV-2 and its variants at present. Therefore, we had contrived a multivalent epitope vaccine of S protein to cope with the problem of virus mutation more effectively. In this study, we took the VOC including Wuhan-Hu-1 and SARS-CoV-2 variants B.1.1.7, B.1.351, P.1, B.1.429, B.1.617.2, etc as the objects of study, and took S proteins of virus strains as the research target. Meanwhile, the immunogenicity of the vaccine was confirmed *in vivo* and *in vitro* study. Theoretically, the vaccine designed by this study has a broader protective effect for SARS-CoV-2 infection.

When recognizing the antigenic peptide presented by APCs or target cells, the T cell receptor (TCR) should recognize both the antigenic peptide and the polymorphic parts of its MHC molecules. Therefore, MHC molecular restriction was fully considered when screening the CD8^+^ and CD4^+^ T-cell epitopes for activating the cellular immunity. In order to expand population coverage of the vaccine, we predicted 27 kinds of HLA-I and 27 kinds of HLA-II T-cell epitopes respectively (60). By comparing the predicted epitope sequences of each virus strain, the common epitopes of different virus strains with the highest frequency were screened and selected. Ultimately, the global population coverage of CTLEs and HTLEs was 93.16% and 99.9% separately in our *rSMEV*. That means the epitopes-based vaccine might be employed by more population in different regions.

To ensure the SARS-CoV-2 vaccine more reliable and effective, we spliced together the chosen epitopes that included 8 CTLEs, 17 HTLEs, 9 LBEs and 4 CBEs by linker sequences. The appropriate linkers could avoid the formation of new epitopes (Linker-epitope) and enhance the presentation of antigen epitopes by ensuring the full exposure of the screened target epitopes (61, 62). Then, the TAT sequence (11aa) was attached to the N-terminal of vaccine construct simultaneously. The TAT sequence can carry macromolecular substances, making it easier to penetrate the cell membrane, so as to promote the phagocytosis of vaccine protein by APCs (36). After the design and construction of the vaccine, the secondary structure of *rSMEV* was analyzed. The secondary structure analysis showed that the vaccine protein had 6.51% Beta turn and 49.51% Random coil. Because the spatial structure of Beta turn and random coil is loose, it is easy to form epitopes. Therefore, the *rSMEV* that containing a large amount of this structure has a good vaccine structural basis.

In view of Rosettafold’s excellent performance in protein tertiary structure prediction (38), we used it to predict the high-quality tertiary structure of *rSMEV*. Meanwhile, the unreliable loops or termini of the predicted 3D structure were reconstructed and refined by the GalaxyRefine Server. The good tertiary structure of *rSMEV* would be used for later molecular docking and MD simulation. We further analyzed the physicochemical properties and allergenicity of *rSMEV.* The physicochemical properties results showed that molecular weight of *rSMEV* was 65 KD. Previous studies have reported that it is reasonable when the molecular weight of vaccine protein is less than 110 KD (56), which hinted that the *rSMEV* was suitable for vaccine. In addition, the analysis of theoretical pI, amino acid acidity, alkalinity, stability, hydrophilicity and allergenicity also displayed that the *rSMEV* was reasonable and safe.

When microorganisms break through the mucosal barrier, TLR can recognize them and initiating the adaptive immune response (63). Because TLR-3 molecule plays an important role in innate immunity against virus, the binding of antigen and TLR-3 can help APCs to present antigen and release local cytokines. For the sake of proving that the *rSMEV* had strong affinity with TLR-3 molecule, we conducted the molecular docking using ClusPro Server. The results showed that lowest energy value in the all outputted *rSMEV*-TLR-3 complexes was −1175.3 kcal/mol. The capacity of the system is lower, the structure of object is more stable (45). Therefore, the structure of *rSMEV*-TLR-3 complex was quite stable. Moreover, the interaction interfaces proved that the binding affinity between *rSMEV* and TLR-3 was ionic and hydrogen bonds mainly. Due to the interaction of ionic bond and hydrogen bond is strong, this also indirectly indicates that the binding between *rSMEV* and TLR-3 is very close (64).

We also used MD simulation to analyze the structural stability and agility of *rSMEV*-TLR-3 complex. The RMSD value represented the structural fluctuation of the overall structure of the vaccine and TLR-3 complex, which can evaluate the stability of the system during interaction. In this part, the floating range of 100 ns RMSD value was about 1 nm, suggesting that the structure of the complex is relatively stable. The RMSF value represented the flexibility of amino acid residues in the docking complex, which can evaluate the agility of each residues in the complex. The results suggested that the agility of receptor and ligand sites after 650 was higher than that of residues 0-650. The average binding free energy of the system calculated by the MMGBSA tool was −88.54 ± 6.91 kcal/mol. Therefore, MD simulation proved that *rSMEV* and TLR-3 docking complex in this study had reasonable structural stability and agility. The above results showed that *rSMEV* can closely bind to TLR-3, and the complex has high stability and agility.

That is more important, we had verified the immunogenicity of the *rSMEV in vivo* and *in vitro.* Firstly, the *rSMEV* can stimulate an excellent immune response confirmed by immune simulation. Then the mice were immunized with *rSMEV* through experiments *in vivo* and *in vitro*. The mice can produce specific ASCs and high levels of IFN-γ and IL-4, the result confirmed that the *rSMEV* can induce cellular and humoral immunity and has satisfied immunogenicity.

In many current studies that based on S proteins to design SARS-CoV-2 vaccine (46–51, 65), there are few studies on designing for multiple variants at the same time. Starting from considering the mutation of SARS-CoV-2, to the restriction of HLA alleles, and then to the binding between vaccine protein and TLR-3 molecule. Our research had made innovative and reasonable design in the above aspects for improving the immunogenicity and applicability of SARS-CoV-2 vaccine. At the same time, we also evaluated the immunogenicity of the vaccine by experiments *in vivo* and *in vitro*. Compared with other studies, our *rSMEV* is more significant in the current perspective. Obviously, the *rSMEV* designed by the study estimated that it may have a high potency against SARS-CoV-2 and its variants theoretically. However, this study did not include other antigens with important immunogenicity of SARS-CoV-2. At the same time, this study still needs to further carry out human clinical trials to ensure the effectiveness of the vaccine. All in all, we provided an effective immunological strategy and mean for the active prevention and control of COVID-19 worldwide.

## Data Availability Statement

The original contributions presented in the study are included in the article/**Supplementary Material**. Further inquiries can be directed to the corresponding authors.

## Ethics Statement

The animal study was reviewed and approved by The Animal Experiment Medical Ethics Committee of the First Affiliated Hospital of Xinjiang Medical University.

## Author Contributions

MY, YZ, YL, ZC, ZWL, JW, and ZNL performed the experiments. MY and YZ wrote and edited the paper. YL, ZWL, JW, and ZNL collated and verified the experimental data. FZ and JD provided experimental design and ideas, and reviewed and modified the paper. All the authors agreed to publish the final version of the paper.

## Funding

This study had been supported by National Natural Science Foundation of China [Grant number: 81960373, 81860352, 31560262] and The key R&D project of Xinjiang Autonomous Region: Research on key technologies of prevention and control for COVID-19 [Project number: 2021B03003-2].

## Conflict of Interest

The authors declare that the research was conducted in the absence of any commercial or financial relationships that could be construed as a potential conflict of interest.

## Publisher’s Note

All claims expressed in this article are solely those of the authors and do not necessarily represent those of their affiliated organizations, or those of the publisher, the editors and the reviewers. Any product that may be evaluated in this article, or claim that may be made by its manufacturer, is not guaranteed or endorsed by the publisher.
