# Supplementary material for: Design of a Recombinant Multivalent Epitope Vaccine Based on SARS-CoV-2 and Its Variants in Immunoinformatics Approaches
Source: Front Immunol. 2022 May 6;13:884433. doi: 10.3389/fimmu.2022.884433 (PMC9120605; doi:10.3389/fimmu.2022.884433)
Supplement: Supplementary file 1 [file DataSheet_1.pdf]

Supplementary Material

1 Supplementary Data

1.1 The amino acid sequence of S proteins

YP_009724390: MFVFLVLLPLVSSQCVNLTTRTQLPPAYTNSFTRGVYYPDKVFRSSVL
HSTQDLFLPFFSNVTWFHAIHVSGTNGTKRFDNPVLPFNDGVYFASTEKSNIIRGWIFGTTLD
SKTQSLLIVNNATNVVIKVCEFQFCNDPFLGVYYHKNNKSWMESEFRVYSSANNCTFEYVS
QPFLMDLEGKQGNFKNLREFVFKNIDGYFKIYSKHTPINLVRDLPQGFSALEPLVDLPIGINIT
RFQTLLALHRSYLTPGDSSSGWTAGAAAYYVGYLQPRTFLLKYNENGTITDAVDCALDPLS
ETKCTLKSFTVEKGIYQTSNFRVQPTESIVRFPNITNLCPFGEVFNATRFASVYAWNRKRISNC
VADYSVLYNSASFSTFKCYGVSPTKLNDLCFTNVYADSFVIRGDEVRQIAPGQTGKIADYNY
KLPDDFTGCVIAWNSNNLDSKVGGNYNYLYRLFRKSNLKPFERDISTEIYQAGSTPCNGVEG
FNCYFPLQSYGFQPTNGVGYQPYRVVVLSFELLHAPATVCGPKKSTNLVKNKCVNFNFNGL
TGTGVLTESNKKFLPFQQFGRDIADTTDAVRDPQTLEILDITPCSFGGVSVITPGTNTSNQVAV
LYQDVNCTEVPVAIHADQLTPTWRVYSTGSNVFQTRAGCLIGAEHVNNSYECDIPIGAGICA
SYQTQTNSPRRARSVASQSIIAYTMSLGAENSVAYSNNSIAIPTNFTISVTTEILPVSMTKTSVD
CTMYICGDSTECSNLLLQYGSFCTQLNRALTGIAVEQDKNTQEVFAQVKQIYKTPPIKDFGG
FNFSQILPDPSKPSKRSFIEDLLFNKVTLADAGFIKQYGDCLGDIAARDLICAQKFNGLTVLPP
LLTDEMIAQYTSALLAGTITSGWTFGAGAALQIPFAMQMAYRFNGIGVTQNVLYENQKLIA
NQFNSAIGKIQDSLSSTASALGKLQDVVNQNAQALNTLVKQLSSNFGAISSVLNDILSRLDKV
EAEVQIDRLITGRLQSLQTYVTQQLIRAAEIRASANLAATKMSECVLGQSKRVDFCGKGYHL
MSFPQSAPHGVVFLHVTYVPAQEKNFTTAPAICHDGKAHFPREGVFVSNGTHWFVTQRNFY
EPQIITTDNTFVSGNCDVVIGIVNNTVYDPLQPELDSFKEELDKYFKNHTSPDVDLGDISGINA
SVVNIQKEIDRLNEVAKNLNESLIDLQELGKYEQYIKWPWYIWLGFIAGLIAIVMVTIMLCC
MTSCCSCLKGCCSCGSCCKFDEDDSEPVLKGVKLHYT

QRX39358: MFVFLVLLPLVSSQCVNLTTRTQLPPAYTNSFTRGVYYPDKVFRSSVLHST
QDLFLPFFSNVTWFHAISGTNGTKRFDNPVLPFNDGVYFASTEKSNIIRGWIFGTTLDSKTQSL
LIVNNATNVVIKVCEFQFCNDPFLGVYHKNNKSWMESEFRVYSSANNCTFEYVSQPFLMDL
EGKQGNFKNLREFVFKNIDGYFKIYSKHTPINLVRDLPQGFSALEPLVDLPIGINITRFQTLLA
LHRSYLTPGDSSSGWTAGAAAYYVGYLQPRTFLLKYNENGTITDAVDCALDPLSETKCTLK

SFTVEKGIYQTSNFRVQPTESIVRFPNITNLCPFGEVFNATRFASVYAWNRKRISNCVADYSV
LYNSASFSTFKCYGVSPTKLNDLCFTNVYADSFVIRGDEVRQIAPGQTGKIADYNYKLPDDF
TGCVIAWNSNNLDSKVGGNYNYLYRLFRKSNLKPFERDISTEIYQAGSTPCNGVEGFNCYFP
LQSYGFQPTYGVGYQPYRVVVLSFELLHAPATVCGPKKSTNLVKNKCVNFNFNGLTGTGVL
TESNKKFLPFQQFGRDIDDTTDAVRDPQTLEILDITPCSFGGVSVITPGTNTSNQVAVLYQGV
NCTEVPVAIHADQLTPTWRVYSTGSNVFQTRAGCLIGAEHVNNSYECDIPIGAGICASYQTQ
TNSHRRARSVASQSIIAYTMSLGAENSVAYSNNSIAIPINFTISVTTEILPVSMTKTSVDCTMYI
CGDSTECSNLLLQYGSFCTQLNRALTGIAVEQDKNTQEVFAQVKQIYKTPPIKDFGGFNFSQI
LPDPSKPSKRSFIEDLLFNKVTLADAGFIKQYGDCLGDIAARDLICAQKFNGLTVLPPLLTDE
MIAQYTSALLAGTITSGWTFGAGAALQIPFAMQMAYRFNGIGVTQNVLYENQKLIANQFNS
AIGKIQDSLSSTASALGKLQDVVNQNAQALNTLVKQLSSNFGAISSVLNDILARLDKVEAEV
QIDRLITGRLQSLQTYVTQQLIRAAEIRASANLAATKMSECVLGQSKRVDFCGKGYHLMSFP
QSAPHGVVFLHVTYVPAQEKNFTTAPAICHDGKAHFPREGVFVSNGTHWFVTQRNFYEPQII
TTHNTFVSGNCDVVIGIVNNTVYDPLQPELDSFKEELDKYFKNHTSPDVDLGDISGINASVVN
IQKEIDRLNEVAKNLNESLIDLQELGKYEQYIKWPWYIWLGFIAGLIAIVMVTIMLCCMTSCC
SCLKGCCSCGSCCKFDEDDSEPVLKGVKLHYT

QRV12312: MFVFLVLLPLVSSQCVNLTTRTQLPPAYTNSFTRGVYYPDKVFRSSVLHST
QDLFLPFFSNVTWFHAIHVSGTNGTKRFANPVLPFNDGVYFASTEKSNIIRGWIFGTTLDSKT
QSLLIVNNATNVVIKVCEFQFCNDPFLGVYYHKNNKSWMESEFRVYSSANNCTFEYVSQPFL
MDLEGKQGNFKNLREFVFKNIDGYFKIYSKHTPINLVRGLPQGFSALEPLVDLPIGINITRFQT
LHRSYLTPGDSSSGWTAGAAAYYVGYLQPRTFLLKYNENGTITDAVDCALDPLSETKCTLK
SFTVEKGIYQTSNFRVQPTESIVRFPNITNLCPFGEVFNATRFASVYAWNRKRISNCVADYSV
LYNSASFSTFKCYGVSPTKLNDLCFTNVYADSFVIRGDEVRQIAPGQTGKIADYNYKLPDDF
TGCVIAWNSNNLDSKVGGNYNYLYRLFRKSNLKPFERDISTEIYQAGSTPCNGVKGFNCYFP
LQSYGFQPTYGVGYQPYRVVVLSFELLHAPATVCGPKKSTNLVKNKCVNFNFNGLTGTGVL
TESNKKFLPFQQFGRDIADTTDAVRDPQTLEILDITPCSFGGVSVITPGTNTSNQVAVLYQGV
NCTEVPVAIHADQLTPTWRVYSTGSNVFQTRAGCLIGAEHVNNSYECDIPIGAGICASYQTQ
TNSPRRARSVASQSIIAYTMSLGVENSVAYSNNSIAIPTNFTISVTTEILPVSMTKTSVDCTMYI
CGDSTECSNLLLQYGSFCTQLNRALTGIAVEQDKNTQEVFAQVKQIYKTPPIKDFGGFNFSQI
LPDPSKPSKRSFIEDLLFNKVTLADAGFIKQYGDCLGDIAARDLICAQKFNGLTVLPPLLTDE
MIAQYTSALLAGTITSGWTFGAGAALQIPFAMQMAYRFNGIGVTQNVLYENQKLIANQFNS
AIGKIQDSLSSTASALGKLQDVVNQNAQALNTLVKQLSSNFGAISSVLNDILSRLDKVEAEV

QIDRLITGRLQSLQTYVTQQLIRAAEIRASANLAATKMSECVLGQSKRVDFCGKGYHLMSFP
QSAPHGVVFLHVTYVPAQEKNFTTAPAICHDGKAHFPREGVFVSNGTHWFVTQRNFYEPQII
TTDNTFVSGNCDVVIGIVNNTVYDPLQPELDSFKEELDKYFKNHTSPDVDLGDISGINASVVN
IQKEIDRLNEVAKNLNESLIDLQELGKYEQYIKWPWYIWLGFIAGLIAIVMVTIMLCCMTSCC
SCLKGCCSCGSCCKFDEDDSEPVLKGVKLHYT

QRX39425: MFVFLVLLPLVSSQCVNFTNRTQLPSAYTNSFTRGVYYPDKVFRSSVLHST
QDLFLPFFSNVTWFHAIHVSGTNGTKRFDNPVLPFNDGVYFASTEKSNIIRGWIFGTTLDSKT
QSLLIVNNATNVVIKVCEFQFCNYPFLGVYYHKNNKSWMESEFRVYSSANNCTFEYVSQPFL
MDLEGKQGNFKNLSEFVFKNIDGYFKIYSKHTPINLVRDLPQGFSALEPLVDLPIGINITRFQT
LLALHRSYLTPGDSSSGWTAGAAAYYVGYLQPRTFLLKYNENGTITDAVDCALDPLSETKC
TLKSFTVEKGIYQTSNFRVQPTESIVRFPNITNLCPFGEVFNATRFASVYAWNRKRISNCVAD
YSVLYNSASFSTFKCYGVSPTKLNDLCFTNVYADSFVIRGDEVRQIAPGQTGTIADYNYKLP
DDFTGCVIAWNSNNLDSKVGGNYNYLYRLFRKSNLKPFERDISTEIYQAGSTPCNGVKGFNC
YFPLQSYGFQPTYGVGYQPYRVVVLSFELLHAPATVCGPKKSTNLVKNKCVNFNFNGLTGT
GVLTESNKKFLPFQQFGRDIADTTDAVRDPQTLEILDITPCSFGGVSVITPGTNTSNQVAVLY
QGVNCTEVPVAIHADQLTPTWRVYSTGSNVFQTRAGCLIGAEYVNNSYECDIPIGAGICASY
QTQTNSPRRARSVASQSIIAYTMSLGAENSVAYSNNSIAIPTNFTISVTTEILPVSMTKTSVDCT
MYICGDSTECSNLLLQYGSFCTQLNRALTGIAVEQDKNTQEVFAQVKQIYKTPPIKDFGGFN
FSQILPDPSKPSKRSFIEDLLFNKVTLADAGFIKQYGDCLGDIAARDLICAQKFNGLTVLPPLL
TDEMIAQYTSALLAGTITSGWTFGAGAALQIPFAMQMAYRFNGIGVTQNVLYENQKLIANQ
FNSAIGKIQDSLSSTASALGKLQDVVNQNAQALNTLVKQLSSNFGAISSVLNDILSRLDKVEA
EVQIDRLITGRLQSLQTYVTQQLIRAAEIRASANLAAIKMSECVLGQSKRVDFCGKGYHLMS
FPQSAPHGVVFLHVTYVPAQEKNFTTAPAICHDGKAHFPREGVFVSNGTHWFVTQRNFYEP
QIITTDNTFVSGNCDVVIGIVNNTVYDPLQPELDSFKEELDKYFKNHTSPDVDLGDISGINASF
VNIQKEIDRLNEVAKNLNESLIDLQELGKYEQYIKWPWYIWLGFIAGLIAIVMVTIMLCCMTS
CCSCLKGCCSCGSCCKFDEDDSEPVLKGVKLHYT

QRW35522: MFVFLVLLPLVSIQCVNLTTRTQLPPAYTNSFTRGVYYPDKVFRSSVLHST
QDLFLPFFSNVTWFHAIHVSGTNGTKRFDNPVLPFNDGVYFASTEKSNIIRGWIFGTTLDSKT
QSLLIVNNATNVVIKVCEFQFCNDPFLGVYYHKNNKSCMESEFRVYSSANNCTFEYVSQPFL
MDLEGKQGNFKNLREFVFKNIDGYFKIYSKHTPINLVRDLPQGFSALEPLVDLPIGINITRFQT
LLALHRSYLTPGDSSSGWTAGAAAYYVGYLQPRIFLLKYNENGTITDAVDCALDPLSETKCT
LKSFTVEKGIYQTSNFRVQPTESIVRFPNITNLCPFGEVFNATRFASVYAWNRKRISNCVADY

SVLYNSASFSTFKCYGVSPTKLNDLCFTNVYADSFVIRGDEVRQIAPGQTGKIADYNYKLPD DFTGCVIAWNSNNLDSKVGGNYNYRYRLFRKSNLKPFERDISTEIYQAGSTPCNGVEGFNCY FPLQSYGFQPTNGVGYQPYRVVVLSFELLHAPATVCGPKKSTNLVKNKCVNFNFNGLTGTG VLTESNKKFLPFQQFGRDIADTTDAVRDPQTLEILDITPCSFGGVSVITPGTNTSNQVAVLYQ GVNCTEVPVAIHADQLTPTWRVYSTGSNVFQTRAGCLIGAEHVNNSYECDIPIGAGICASYQ TQTNSPRRARSVASQSIIAYTMSLGAENSVAYSNNSIAIPTNFTISVTTEILPVSMTKTSVDCT MYICGDSTECSNLLLQYGSFCTQLNRALTGIAVEQDKNTQEVFAQVKQIYKTPPIKDFGGFN FSQILPDPSKPSKRSFIEDLLFNKVTLADAGFIKQYGDCLGDIAARDLICAQKFNGLTVLPPLL TDEMIAQYTSALLAGTITSGWTFGAGAALQIPFAMQMAYRFNGIGVTQNVLYENQKLIANQ FNSAIGKIQDSLSSTASALGKLQDVVNQNAQALNTLVKQLSSNFGAISSVLNDILSRLDKVEA EVQIDRLITGRLQSLQTYVTQQLIRAAEIRASANLAATKMSECVLGQSKRVDFCGKGYHLMS FPQSAPHGVVFLHVTYVPAQEKNFTTAPAICHDGKAHFPREGVFVSNGTHWFVTQRNFYEP QIITTDNTFVSGNCDVVIGIVNNTVYDPLQPELDSFKEELDKYFKNHTSPDVDLGDISGINASV VNIQKEIDRLNEVAKNLNESLIDLQELGKYEQYIKWPWYIWLGFIAGLIAIVMVTIMLCCMTS CCSCLKGCCSCGSCCKFDEDDSEPVLKGVKLHYT

QXF88313: MFVFLVLLPLVSSQCVNLRTRTQLPPAYTNSFTRGVYYPDKVFRSSVLHST QDLFLPFFSNVTWFHAIHVSGTNGTKRFDNPVLPFNDGVYFASTEKSNIIRGWIFGTTLDSKT QSLLIVNNATNVVIKVCEFQFCNDPFLDVYYHKNNKSWMESGVYSSANNCTFEYVSQPFLM DLEGKQGNFKNLREFVFKNIDGYFKIYSKHTPINLVRDLPQGFSALEPLVDLPIGINITRFQTL LALHRSYLTPGDSSSGWTAGAAAYYVGYLQPRTFLLKYNENGTITDAVDCALDPLSETKCT LKSFTVEKGIYQTSNFRVQPTESIVRFPNITNLCPFGEVFNATRFASVYAWNRKRISNCVADY SVLYNSASFSTFKCYGVSPTKLNDLCFTNVYADSFVIRGDEVRQIAPGQTGKIADYNYKLPD DFTGCVIAWNSNNLDSKVGGNYNYRYRLFRKSNLKPFERDISTEIYQAGSKPCNGVEGFNCY FPLQSYGFQPTNGVGYQPYRVVVLSFELLHAPATVCGPKKSTNLVKNKCVNFNFNGLTGTG VLTESNKKFLPFQQFGRDIADTTDAVRDPQTLEILDITPCSFGGVSVITPGTNTSNQVAVLYQ GVNCTEVPVAIHADQLTPTWRVYSTGSNVFQTRAGCLIGAEHVNNSYECDIPIGAGICASYQ TQTNSRRRARSVASQSIIAYTMSLGAENSVAYSNNSIAIPTNFTISVTTEILPVSMTKTSVDCT MYICGDSTECSNLLLQYGSFCTQLNRALTGIAVEQDKNTQEVFAQVKQIYKTPPIKDFGGFN FSQILPDPSKPSKRSFIEDLLFNKVTLADAGFIKQYGDCLGDIAARDLICAQKFNGLTVLPPLL TDEMIAQYTSALLAGTITSGWTFGAGAALQIPFAMQMAYRFNGIGVTQNVLYENQKLIANQ FNSAIGKIQDSLSSTASALGKLQNVVNQNAQALNTLVKQLSSNFGAISSVLNDILSRLDKVEA EVQIDRLITGRLQSLQTYVTQQLIRAAEIRASANLAATKMSECVLGQSKRVDFCGKGYHLMS

FPQSAPHGVVFLHVTYVPAQEKNFTTAPAICHDGKAHFPREGVFVSNGTHWFVTQRNFYEP
QIITTDNTFVSGNCDVVIGIVNNTVYDPLQPELDSFKEELDKYFKNHTSPDVDLGDISGINASV
VNIQKEIDRLNEVAKNLNESLIDLQELGKYEQYIKWPWYIWLGFIAGLIAIVMVTIMLCCMTS
CCSCLKGCCSCGSCCKFDEDDSEPVLKGVKLHYT

1.2 The DNA sequence of *rSMEV*

5'-ACCACCCGTACCCAGCTGCCGCCGGCTTACGCTGCTTACCGTACCCGTACCCAGCTGC
CGCCGGCTTACGCTGCTTACCTGCCGTTCTTCTCTAACGTTACCTGGTTCGCTGCTTACCT
GCCGATCGGTATCAACATCACCCGTTTCGCTGCTTACTTCCCGAACATCACCAACCTGTG
CCCGTTCGCTGCTTACCTGCAGTCTTACGGTTTCCAGCCGACCTACGCTGCTTACCAGCC
GTACCGTGTTGTTGTTCTGTCTTTCGCTGCTTACAACACCTCTAACCAGGTTGCTGTTCTG
TACGCTGCTTACATCGGTATCAACATCACCCGTTTCCAGACCCTGCACCGTTCTTACGGT
CCGGGTCCGGGTAACGACGGTGTTTACTTCGCTTCTACCGAAAAATCTAACATCATCGGT
CCGGGTCCGGGTGGTATCAACATAACTAGGTTCCAGACCCTGCATCGTTCTTACCTGGGT
CCGGGTCCGGGTGTTGAAAAAGGTATCTACCAGACCTCTAACTTCCGTGTTCAGCCGGG
TCCGGGTCCGGGTGAAAAAGGTATCTACCAGACCTCTAACTTCCGTGTTCAGCCGACCG
GTCCGGGTCCGGGTAAAGGTATCTACCAGACCTCTAACTTCCGTGTTCAGCCGACCGAA
GGTCCGGGTCCGGGTCAGACCTCTAACTTCCGTGTTCAGCCGACCGAATCTATCGTTCGT
GGTCCGGGTCCGGGTACCTCTAACTTCCGTGTTCAGCCGACCGAATCTATAGTTAGGTTC
GGTCCGGGTCCAGGTAACCGTGCTCTGACCGGTATCGCTGTTGAACAGGACAAAAACAC
AGGTCCAGGCCCGGGCTTCGGCGGCTTCAACTTCTCTCAGATCCTGCCGGACCCGTCTAA
AGGTCCGGGTCCGGGTCGTGCTGCTGAAATCCGTGCTTCTGCTAACCTGGCTGCTACCAA
AGGTCCGGGTCCGGGTCGTGCTGCTGAAATCCGTGCTTCTGCTAACCTGGCTGCTATCAA
AGGTCCGGGTCCGGGTGCTGCTGAAATCCGTGCTTCTGCTAACCTGGCTGCTACCAAAAT
GGGTCCGGGTCCGGGTGCTGCTGAAATCCGTGCTTCTGCTAACCTGGCTGCTATCAAAAT
GGGTCCGGGTCCGGGTGAACCGCAGATCATCACCACCGACAACACCTTCGTTTCTGGTA
ACGGTCCGGGTCCGGGTTACGAACCGCAGATCATCACCACCGACAACACCTTCGTTTCT
GGTGGTCCGGGTCCGGGTGAACCGCAGATCATCACCACCGACAACACCTTCGTTTCTGG
TAACGGTCCGGGTCCGGGTTGCGTTAACCTGACCACCCGTACCCAGCTGCCGCCGAAAA
AAGTTAGGGGTCTGCCACAGGGCTTCTCTGCTCTGGAGAAAAAACGTCAGATCGCTCCG
GGTCAGACCGGTAAAATCGCTAAAAAAGTTCGTCAGATCGCTCCGGGTCAGACCGGTAC
CATCAAAAAAAACTTCAACTTCAACGGTCTGACCGGTACCGGTGTTAAAAAATCTAAAG
TTGGTGGTAACTACAACTACCGTTACCGTAAAAAAGAATGCGACATCCCGATCGGTGCT

GGTATCTGCGCTAAAAAACACGTTACCTACGTTCCGGCTCAGGAAAAAAACTTCAAAAA
AAACAACACCGTTTACGACCCGCTGCAGCCGGAACTGAAAAAATACGACCCGCTGCAGC
CGGAACTGGACTCTAAAAAAAACTCTGTTGCTAAAAAAAAAAAACCACACCTCTCCGAAA
AAAAACCACACCTCTCCGAAAAAAACCGGTGCTCTGCTGGCTGCTGGTGCTGCTGCT-**3’**

2 Supplementary Figures

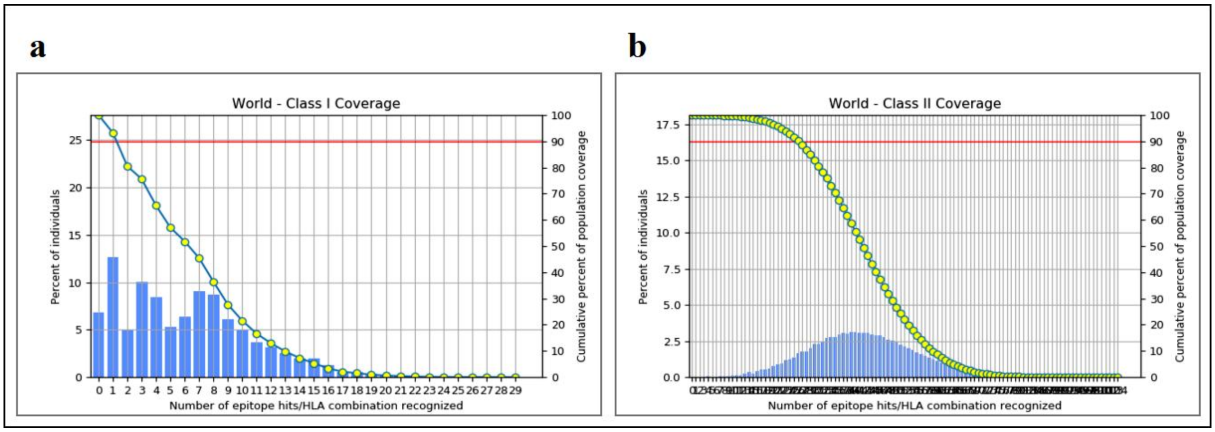

Supplementary Figure 1. The evaluation of *rSMEV* population coverage.

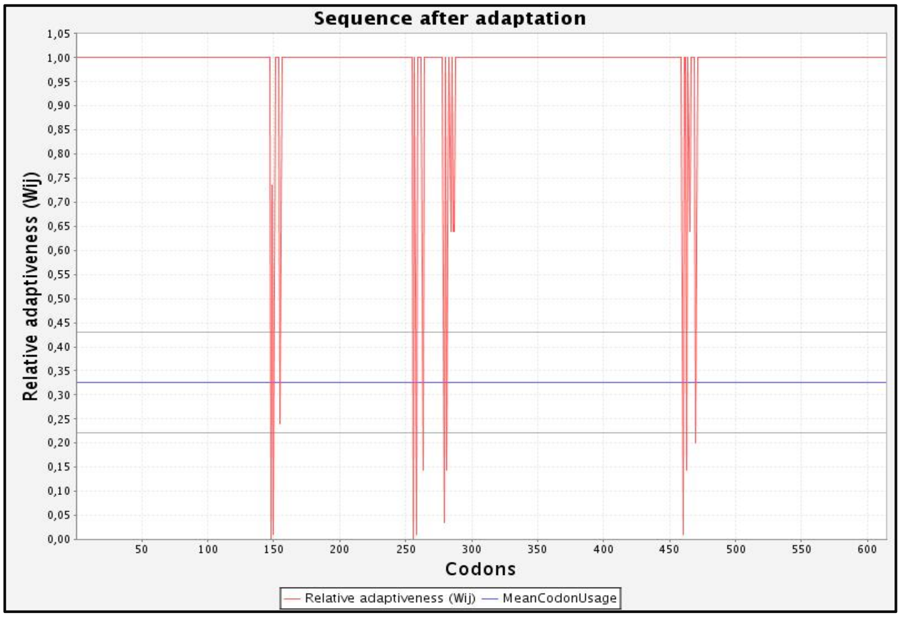

Supplementary Figure 2. The codon optimization of *rSMEV*.

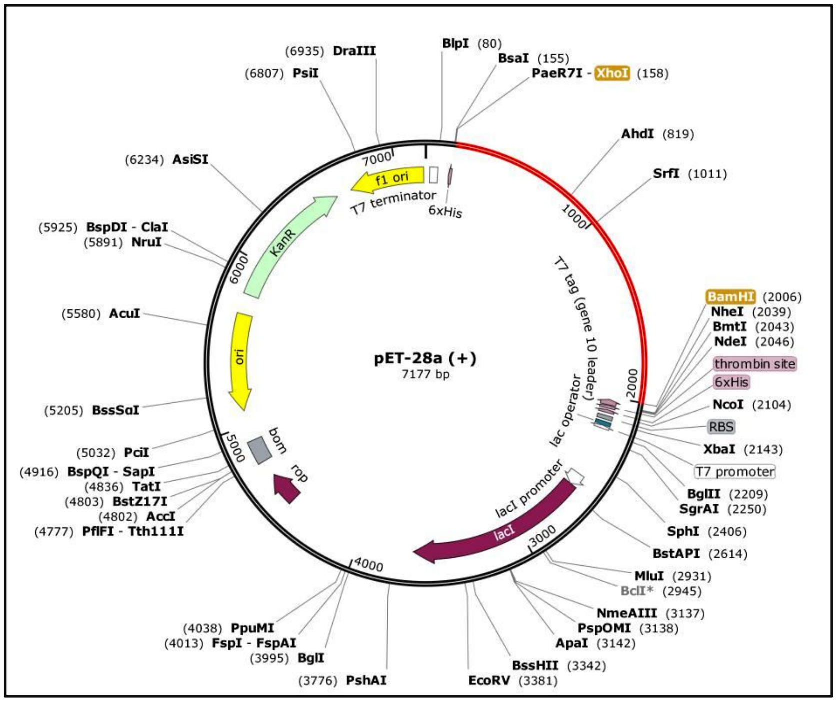

Supplementary Figure 3. The silico cloning of *rSMEV*.

3 Supplementary Tables

Supplementary Table 1. The CTL epitopes of vaccine construction

No.	Epitope sequence	Antigenicity value [a]	Variant epitope [b]	Protein	Lineage
1	TTRTQLPPAY	1.2001	RTRTQLPPAY	S	Wuhan-Hu-1
2	RTRTQLPPAY	1.2188	LQSYGFQPTY		B.1.1.7
3	YTNSFTRGVY	-0.3695			B.1.351
4	LPFFSNVTWF	0.6567			P.1
5	SSANNCTFEY	-0.0845			B.1.429
6	CTFEYVSQPF	0.2678			B.1.617.2
7	LPIGINITRF	1.3027			
8	CTLKSFTVEK	0.0174			
9	FPNITNLCPF	1.3964			
10	YSVLYNSASF	0.1881			
11	LQSYGFQPTY	0.8035			
12	QPYRVVVLSF	0.8458			
13	NTSNQVAVLY	0.4403			
14	RVYSTGSNVF	-0.1018			
15	SVASQSIIAY	0.2707			
16	FVSNGTHWFV	-0.1079			

[a] *When the antigenicity value＞0.4 (default threshold), the epitopes were used to construct vaccine.*
[b] *The antigen epitopes that appeared only in SARS-CoV-2 variants.*

Supplementary Table 2. The HTL epitopes of vaccine construction

No.	Peptide sequence	Antigenicity value [a]	Variant epitope [b]	Protein	Lineage
1	PDKVFRSSVLHSTQD	-0.0726	IGINITRFQTLHRSY	S	Wuhan-Hu-1
2	DKVFRSSVLHSTQDL	-0.0738	NDGVYFASTEKSNII		B.1.1.7
3	IGINITRFQTLHRSY	0.8700	GINITRFQTLHRSYL		B.1.351
4	NDGVYFASTEKSNII	0.4277	ENQKLIANQFNSAIG		P.1
5	HTPINLVRDLPQGFS	0.3970	RAAEIRASANLAAIK		B.1.429
6	GINITRFQTLHRSYL	0.6001	AAEIRASANLAAIKM		B.1.617.2
7	VEKGIYQTSNFRVQP	0.7959	YEPQIITTDNTFVSG		
8	EKGIYQTSNFRVQPT	0.9243			
9	KGIYQTSNFRVQPTE	0.8838			
10	QTSNFRVQPTESIVR	0.4885			
11	TSNFRVQPTESIVRF	0.4574			
12	NRALTGIAVEQDKNT	0.5298			
13	FGGFNFSQILPDPSK	0.4404			
14	NQKLIANQFNSAIGK	-0.1261			
15	ENQKLIANQFNSAIG	0.0952			
16	RAAEIRASANLAATK	0.5709			
17	RAAEIRASANLAAIK	0.5150			
18	AAEIRASANLAATKM	0.7125			
19	AAEIRASANLAAIKM	0.7249			
20	EPQIITTDNTFVSGN	0.4586			
21	YEPQIITTDNTFVSG	0.5066			

[a] *When the antigenicity value＞0.4 (default threshold), the epitopes were used to construct vaccine.*
[b] *The antigen epitopes that appeared only in SARS-CoV-2 variants.*

Supplementary Table 3. The linear B-cell epitopes of vaccine construction

No.	Peptide sequence	Antigenicity value [a]	Variant epitope [b]	Protein	Lineage involved
1	CVNLTTRTQLPP	1.6490	VRGLPQGFSALE	S	Wuhan-Hu-1
2	TRGVYYPDKVFR	0.3643	VRQIAPGQTGTI		B.1.1.7
3	STQDLFLPFFSN	0.1041	NFNFNGLTGTGV		B.1.351
4	ASTEKSNIIRGW	-0.5092	GFIKQYGDCLGD		P.1
5	VRGLPQGFSALE	0.7372			B.1.429
6	TPGDSSSGWTAG	0.0746			B.1.617.2
7	PTESIVRFPNIT	-0.0307			
8	RQIAPGQTGKIA	1.4558			
9	VRQIAPGQTGTI	1.0477			
10	NFNFNGLTGTGV	1.3547			
11	SKVGGNYNYRYR	1.3631			
12	VITPGTNTSNQV	0.3818			
13	ECDIPIGAGICA	0.5970			
14	YQTQTNSPRRAR	0.0760			
15	YKTPPIKDFGGF	-0.3433			
16	GFIKQYGDCLGD	-0.3836			
17	HVTYVPAQEKNF	1.1353			
18	ITTDNTFVSGNC	0.1449			
19	NNTVYDPLQPEL	0.4440			
20	CGSCCKFDEDDS	0.3274			

[a] *When the antigenicity value ＞0.4 (default threshold), the epitopes were used to construct vaccine.*
[b] *The antigen epitopes that appeared only in SARS-CoV-2 variants.*

Supplementary Table 4. The conformational B-cell epitopes of vaccine construction

No.	Peptide sequence	Score	Lineage
1	Y1138, D1139, P1140, L1141, Q1142, P1143, E1144, L1145, D1146, S1147	0.964	Wuhan-Hu-1
2	N700, S701, V702, A703	0.611	B.1.1.7
3	K1154, N1155, H1156, T1157, S1158, P1159	0.997	B.1.351
4	N1158, H1159, T1160, S1161, P1162	0.997	P.1
5	N1158, H1159, T1160, S1161, P1162	0.997	B.1.429
6	N1156, H1157, T1158, S1159, P1160	0.997	B.1.617.2

Supplementary Table 5. HLA-I alleles used in screening CTL epitopes

NO.	Epitopes	Alleles involved [a]
1	TTRTQLPPAY	HLA-A*01:01, HLA-A*26:01, HLA-A*30:01, HLA-A*30:02
2	RTRTQLPPAY	HLA-A*01:01, HLA-A*03:01, HLA-A*30:01, HLA-A*30:02, HLA-B*57:01
3	YTNSFTRGVY	HLA-A*01:01, HLA-A*26:01, HLA-A*30:02, HLA-B*15:01, HLA-B*35:01
4	LPFFSNVTWF	HLA-B*07:02, HLA-B*35:01, HLA-B*51:01, HLA-B*53:01
5	SSANNCTFEY	HLA-A*01:01, HLA-A*11:01, HLA-A*26:01, HLA-A*30:02, HLA-B*58:01
6	CTFEYVSQPF	HLA-A*26:01, HLA-A*32:01, HLA-B*15:01, HLA-B*35:01, HLA-B*53:01, HLA-B*57:01, HLA-B*58:01
7	LPIGINITRF	HLA-B*07:02, HLA-B*35:01, HLA-B*51:01, HLA-B*53:01
8	CTLKSFTVEK	HLA-A*03:01, HLA-A*11:01, HLA-A*30:01, HLA-A*68:01
9	FPNITNLCPF	HLA-B*07:02, HLA-B*35:01, HLA-B*51:01, HLA-B*53:01
10	YSVLYNSASF	HLA-A*26:01, HLA-B*15:01, HLA-B*35:01, HLA-B*53:01, HLA-B*57:01
11	LQSYGFQPTY	HLA-A*01:01, HLA-A*30:02, HLA-B*15:01, HLA-B*44:02, HLA-B*44:03
12	QPYRVVVLSF	HLA-B*07:02, HLA-B*35:01, HLA-B*51:01, HLA-B*53:01
13	NTSNQVAVLY	HLA-A*01:01, HLA-A*26:01, HLA-A*30:02, HLA-A*68:01
14	RVYSTGSNVF	HLA-A*30:02, HLA-A*32:01, HLA-B*07:02, HLA-B*15:01, HLA-B*57:01, HLA-B*58:01
15	SVASQSIIAY	HLA-A*01:01, HLA-A*03:01, HLA-A*11:01, HLA-A*26:01, HLA-A*30:02, HLA-B*15:01, HLA-B*35:01
16	FVSNGTHWFV	HLA-A*02:01, HLA-A*02:03, HLA-A*02:06, HLA-A*68:02

[a] *The alleles of CTL epitopes in the rSMEV, they were derived from 27 HLA-I alleles used to predict CTL epitopes.*

Supplementary Table 6. HLA-II alleles used in screening HTL epitopes

NO.	Epitopes	Alleles involved [a]
1	PDKVFRSSVLHSTQD	HLA-DRB4*01:01, HLA-DQA1*05:01/DQB1*03:01, HLA-DQA1*04:01/DQB1*04:02, HLA-DQA1*01:02/DQB1*06:02, HLA-DPA1*02:01/DPB1*01:01, HLA-DPA1*01:03/DPB1*02:01, HLA-DPA1*01:03/DPB1*04:01, HLA-DPA1*03:01/DPB1*04:02, HLA-DPA1*02:01/DPB1*05:01, HLA-DPA1*02:01/DPB1*14:01
2	DKVFRSSVLHSTQDL	HLA-DQA1*05:01/DQB1*03:01, HLA-DQA1*01:02/DQB1*06:02, HLA-DPA1*02:01/DPB1*01:01, HLA-DPA1*01:03/DPB1*02:01, HLA-DPA1*01:03/DPB1*04:01, HLA-DPA1*03:01/DPB1*04:02, HLA-DPA1*02:01/DPB1*05:01, HLA-DPA1*02:01/DPB1*14:01
3	IGINITRFQTLHRSY	HLA-DRB1*15:01, HLA-DPA1*02:01/DPB1*01:01, HLA-DPA1*01:03/DPB1*02:01, HLA-DPA1*01:03/DPB1*04:01, HLA-DPA1*03:01/DPB1*04:02, HLA-DPA1*02:01/DPB1*05:01
4	NDGVYFASTEKSNII	HLA-DRB1*04:01, HLA-DRB4*01:01, HLA-DQA1*05:01/DQB1*02:01, HLA-DQA1*05:01/DQB1*03:01, HLA-DQA1*03:01/DQB1*03:02, HLA-DQA1*04:01/DQB1*04:02
5	HTPINLVRDLPQGFS	HLA-DRB1*03:01, HLA-DRB1*04:01, HLA-DRB1*04:05, HLA-DRB3*01:01, HLA-DRB4*01:01, HLA-DQA1*03:01/DQB1*03:02, HLA-DQA1*01:01/DQB1*05:01
6	GINITRFQTLHRSYL	HLA-DRB1*15:01, HLA-DRB5*01:01, HLA-DPA1*02:01/DPB1*01:01, HLA-DPA1*01:03/DPB1*02:01, HLA-DPA1*01:03/DPB1*04:01, HLA-DPA1*03:01/DPB1*04:02, HLA-DPA1*02:01/DPB1*05:01
7	VEKGIYQTSNFRVQP	HLA-DRB1*07:01, HLA-DRB1*09:01, HLA-DRB1*12:01, HLA-DRB1*13:02, HLA-DRB1*15:01, HLA-DQA1*01:01/DQB1*05:01, HLA-DPA1*02:01/DPB1*01:01, HLA-DPA1*01:03/DPB1*02:01, HLA-DPA1*01:03/DPB1*04:01, HLA-DPA1*03:01/DPB1*04:02, HLA-DPA1*02:01/DPB1*05:01
8	EKGIYQTSNFRVQPT	HLA-DRB1*07:01, HLA-DRB1*09:01, HLA-DRB1*12:01, HLA-DRB1*13:02, HLA-DRB1*15:01, HLA-DQA1*01:01/DQB1*05:01, HLA-DPA1*02:01/DPB1*01:01, HLA-DPA1*01:03/DPB1*02:01, HLA-DPA1*01:03/DPB1*04:01, HLA-DPA1*03:01/DPB1*04:02, HLA-DPA1*02:01/DPB1*05:01
9	KGIYQTSNFRVQPTE	HLA-DRB1*07:01, HLA-DRB1*13:02, HLA-DRB1*15:01, HLA-DPA1*02:01/DPB1*01:01, HLA-DPA1*01:03/DPB1*02:01, HLA-DPA1*01:03/DPB1*04:01, HLA-DPA1*03:01/DPB1*04:02, HLA-DPA1*02:01/DPB1*05:01,
10	QTSNFRVQPTESIVR	HLA-DRB1*01:01, HLA-DRB1*04:01, HLA-DRB1*04:05, HLA-DRB1*13:02, HLA-DRB3*01:01, HLA-DRB3*02:02, HLA-DRB4*01:01, HLA-DRB5*01:01, HLA-DPA1*02:01/DPB1*01:01, HLA-DPA1*03:01/DPB1*04:02
11	TSNFRVQPTESIVRF	HLA-DRB1*01:01, HLA-DRB1*04:01, HLA-DRB1*04:05, HLA-DRB1*13:02, HLA-DRB3*01:01, HLA-DRB3*02:02, HLA-DRB4*01:01, HLA-DRB5*01:01, HLA-DPA1*02:01/DPB1*01:01, HLA-DPA1*03:01/DPB1*04:02
12	NRALTGIAVEQDKNT	HLA-DRB1*04:05, HLA-DRB4*01:01, HLA-DQA1*05:01/DQB1*02:01, HLA-DQA1*03:01/DQB1*03:02, HLA-DQA1*04:01/DQB1*04:02, HLA-DQA1*01:01/DQB1*05:01, HLA-DQA1*01:02/DQB1*06:02
13	FGGFNFSQILPDPSK	HLA-DRB1*04:05, HLA-DRB1*07:01, HLA-DRB1*09:01, HLA-DQA1*05:01/DQB1*02:01, HLA-DQA1*05:01/DQB1*03:01, HLA-DQA1*03:01/DQB1*03:02, HLA-DQA1*04:01/DQB1*04:02, HLA-DPA1*02:01/DPB1*01:01, HLA-DPA1*01:03/DPB1*04:01, HLA-DPA1*03:01/DPB1*04:02
14	NQKLIANQFNSAIGK	HLA-DRB1*13:02, HLA-DRB3*02:02, HLA-DPA1*02:01/DPB1*01:01, HLA-DPA1*01:03/DPB1*02:01, HLA-DPA1*01:03/DPB1*04:01, HLA-DPA1*03:01/DPB1*04:02, HLA-DPA1*02:01/DPB1*05:01
15	ENQKLIANQFNSAIG	HLA-DPA1*02:01/DPB1*01:01, HLA-DPA1*01:03/DPB1*02:01, HLA-DPA1*01:03/DPB1*04:01, HLA-DPA1*03:01/DPB1*04:02, HLA-DPA1*02:01/DPB1*05:01
16	RAAEIRASANLAATK	HLA-DRB1*04:01, HLA-DRB1*08:02, HLA-DRB1*13:02, HLA-DRB1*15:01, HLA-DRB3*02:02, HLA-DRB4*01:01, HLA-DPA1*02:01/DPB1*14:01,
17	RAAEIRASANLAAIK	HLA-DRB1*04:01, HLA-DRB1*08:02, HLA-DRB1*13:02, HLA-DRB1*15:01, HLA-DRB3*02:02, HLA-DRB4*01:01, HLA-DPA1*02:01/DPB1*14:01,
18	AAEIRASANLAATKM	HLA-DRB1*04:01, HLA-DRB1*08:02, HLA-DRB1*13:02, HLA-DRB1*15:01, HLA-DRB3*02:02, HLA-DRB4*01:01, HLA-DPA1*02:01/DPB1*14:01
19	AAEIRASANLAAIKM	HLA-DRB1*04:01, HLA-DRB1*08:02, HLA-DRB1*13:02, HLA-DRB1*15:01, HLA-DRB3*02:02, HLA-DRB4*01:01, HLA-DPA1*02:01/DPB1*14:01
20	EPQIITTDNTFVSGN	HLA-DRB1*03:01, HLA-DRB1*04:01, HLA-DRB1*07:01, HLA-DRB1*13:02, HLA-DRB3*01:01, HLA-DQA1*05:01/DQB1*02:01, HLA-DQA1*03:01/DQB1*03:02,
21	YEPQIITTDNTFVSG	HLA-DRB1*03:01, HLA-DRB1*04:01, HLA-DRB1*07:01, HLA-DRB1*13:02, HLA-DRB3*01:01, HLA-DQA1*05:01/DQB1*02:01, HLA-DQA1*03:01/DQB1*03:02,
22	EPQIITTDNTFVSGN	HLA-DRB1*03:01, HLA-DRB1*04:01, HLA-DRB1*07:01, HLA-DRB1*13:02, HLA-DRB3*01:01, HLA-DQA1*05:01/DQB1*02:01, HLA-DQA1*03:01/DQB1*03:02

[a] *The alleles of HTL epitopes in the rSMEV, they were derived from 27 HLA-II alleles used to predict HTL epitopes.*

Supplementary Table 7. The result of molecular docking between TLR-3 and *rSMEV*

Cluster	Members	Representative	Weighted Score
0	44	Lowest Energy	−1175.3
1	43	Lowest Energy	−1145.5
2	34	Lowest Energy	−1174.1
3	29	Lowest Energy	−1187.9
4	28	Lowest Energy	−1454.2
5	26	Lowest Energy	−1221.2
6	21	Lowest Energy	−1182.5
7	21	Lowest Energy	−1065.4
8	20	Lowest Energy	−1160.5
9	19	Lowest Energy	−1154.0
10	18	Lowest Energy	−1129.7
11	17	Lowest Energy	−1098.6
12	16	Lowest Energy	−1136.0
13	16	Lowest Energy	−1096.1
14	16	Lowest Energy	−1196.5
15	15	Lowest Energy	−1110.3
16	14	Lowest Energy	−1197.3
17	14	Lowest Energy	−1204.9
18	14	Lowest Energy	−1173.4
19	13	Lowest Energy	−1130.3
20	13	Lowest Energy	−1169.6
21	12	Lowest Energy	−1141.4
22	12	Lowest Energy	−1167.1
23	12	Lowest Energy	−1269.1
24	12	Lowest Energy	−1176.0
25	11	Lowest Energy	−1089.8
26	11	Lowest Energy	−1156.4
27	11	Lowest Energy	−1064.0
28	11	Lowest Energy	−1059.0
29	10	Lowest Energy	−1161.8

Supplementary Table 8. The binding free energy detected by MMGBSA tool

**Energy Component**	**Average**	**Std. Dev.**	**Std. Err. of Mean**
BOND	0.0000	0.0000	0.0000
ANGLE	0.0000	0.0000	0.0000
DIHED	-0.0015	0.0045	0.0011
VDWAALS	-109.1011	5.9343	1.4836
EEL	-301.7082	27.5595	6.8899
1-4 VDW	0.0000	0.0001	0.0000
1-4 EEL	0.0000	0.0001	0.0000
EGB	346.4172	24.5425	6.1356
ESURF	-18.1701	0.7439	0.1860
DELTA G gas	-409.7108	23.8453	5.9613
DELTA G solv	328.2241	24.7952	6.1988
DELTA TOTAL	-88.5437	6.9146	1.7287
